# Muscarinic receptor agonist-induced βPix binding to β-catenin promotes colon neoplasia

**DOI:** 10.1038/s41598-023-44158-8

**Published:** 2023-10-07

**Authors:** Kunrong Cheng, Ahmed Chahdi, Shannon M. Larabee, Mazen Tolaymat, Margaret H. Sundel, Cinthia B. Drachenberg, Min Zhan, Shien Hu, Anan H. Said, Aaron C. Shang, Guofeng Xie, Madeline Alizadeh, Natalia Sampaio Moura, Andrea C. Bafford, Richelle T. Williams, Nader N. Hanna, Jean-Pierre Raufman

**Affiliations:** 1 VA Maryland Healthcare System, Baltimore, MD 21201 USA; 2 Department of Medicine, Division of Gastroenterology and Hepatology, University of Maryland School of Medicine, Baltimore, MD 21201 USA; 3 Department of Surgery, University of Maryland School of Medicine, Baltimore, MD 21201 USA; 4 Department of Pathology, University of Maryland School of Medicine, Baltimore, MD 21201 USA; 5 Department of Epidemiology and Public Health, University of Maryland School of Medicine, Baltimore, MD 21201 USA; 6 Marlene and Stewart Greenebaum Cancer Center, University of Maryland School of Medicine, Baltimore, MD 21201 USA; 7 The Institute for Genome Sciences, University of Maryland School of Medicine, Baltimore, MD 20201 USA; 8 Department of Biochemistry and Molecular Biology, University of Maryland School of Medicine, Baltimore, MD 21201 USA

**Keywords:** Colon cancer, Growth factor signalling

## Abstract

M_3_ muscarinic receptors (M_3_R) modulate β-catenin signaling and colon neoplasia. CDC42/RAC guanine nucleotide exchange factor, βPix, binds to β-catenin in colon cancer cells, augmenting β-catenin transcriptional activity. Using in silico, in vitro, and in vivo approaches, we explored whether these actions are regulated by M_3_R. At the invasive fronts of murine and human colon cancers, we detected co-localized nuclear expression of βPix and β-catenin in stem cells overexpressing M_3_R. Using immunohistochemistry, immunoprecipitation, proximity ligand, and fluorescent cell sorting assays in human tissues and established and primary human colon cancer cell cultures, we detected time-dependent M_3_R agonist-induced cytoplasmic and nuclear association of βPix with β-catenin. βPix knockdown attenuated M_3_R agonist-induced human colon cancer cell proliferation, migration, invasion, and expression of *PTGS2*, the gene encoding cyclooxygenase-2, a key player in colon neoplasia. Overexpressing βPix dose-dependently augmented β-catenin binding to the transcription factor TCF4. In a murine model of sporadic colon cancer, advanced neoplasia was attenuated in conditional knockout mice with intestinal epithelial cell deficiency of βPix. Expression levels of β-catenin target genes and proteins relevant to colon neoplasia, including *c-Myc* and *Ptgs2,* were reduced in colon tumors from βPix-deficient conditional knockout mice. Targeting the M_3_R/βPix/β-catenin axis may have therapeutic potential.

## Introduction

In quiescent intestinal epithelial cells, cytosolic β-catenin is sequestered in a multi-protein complex and targeted for degradation. In ~ 90% of colon cancers APC or β-catenin mutations destabilize β-catenin destruction complexes, freeing β-catenin to translocate to the cell nucleus. As a transcriptional coactivator of TCF4, nuclear β-catenin induces transcription of genes that promote neoplasia, e.g., prostaglandin-endoperoxide synthase 2 [*PTGS2*, *cyclooxygenase2* (*COX2*)]. In colon cancer cells, muscarinic receptor (MR) activation augments β-catenin signaling^1^. Of five MR subtypes, *CHRM3*, a conditional oncogene encoding M_3_R^2^, is overexpressed in 60–80% of colon cancers^3–5^. Post-M_3_R signaling selectively induces genes promoting cell proliferation, migration, and invasion^6–13^. Interconnecting mechanisms involving activation of protein kinase C-α (PKC-α) and transactivation of epidermal growth factor receptors (EGFR) mediate post-M_3_R signaling. M_3_R deficiency attenuates murine intestinal neoplasia^1,14^. Attenuated nuclear accumulation of β-catenin and intestinal tumor formation in M_3_R-deficient *Apc*^*Min/*+^ mice^1,15^, led us to explore functional crosstalk between M_3_R and β-catenin signaling.

Mechanisms whereby Class A guanine nucleotide-binding protein-coupled receptors (GPCRs) like M_3_R modulate β-catenin signaling are incompletely understood^16,17^. We surmised that guanine nucleotide exchange factor (GEF) intermediaries between GPCR activation and cell signaling might play a role. Rho GEF GTPases, which act as switches shuttling between inactive GDP-bound and active GTP-bound proteins, are frequently overexpressed and activated in cancer^18^. We focused on βPak-interacting exchange factor (βPix), a Rho family GEF for Cdc42/Rac1^19,20^. Rac1 regulates cytoskeletal dynamics, cell polarity, migration, and adhesion^21–23^, as well as β-catenin activity and nuclear translocation^24,25^; Rac1 expression and activation are increased in colon cancer^26,27^ and Tiam1, another Rac GEF, modulates canonical β-catenin signaling^28^. Both β-catenin and βPix integrate signals that control cell adhesion and cytoskeletal reorganization^29–33^.

Previous work provides a conceptual framework whereby GPCRs, like M_3_R, can activate GEFs^34^; for example, G_αq_ subunits are capable of activating RhoGEFs like p63RhoGEF, Trio, and Kalirin^35^. Moreover, as we reported for M_3_R^5^, βPix is overexpressed in human colon cancer cell lines^36,37^ and, like M_3_R overexpression^5^, βPix overexpression predicts the presence of colon cancer metastases^36^. These considerations and our previous finding that βPix binds β-catenin in human colon cancer cells and stimulates β-catenin transcriptional activity and cell proliferation^38^, led us to test the possibility that this βPix/β-catenin interaction is regulated, at least in part, by M_3_R activation, with functional consequences that identify this nexus as a novel therapeutic target in colon cancer.

## Results

### βPix and β-catenin colocalize in nuclei of colon cancer cells overexpressing M_3_R

**Figure 1:**
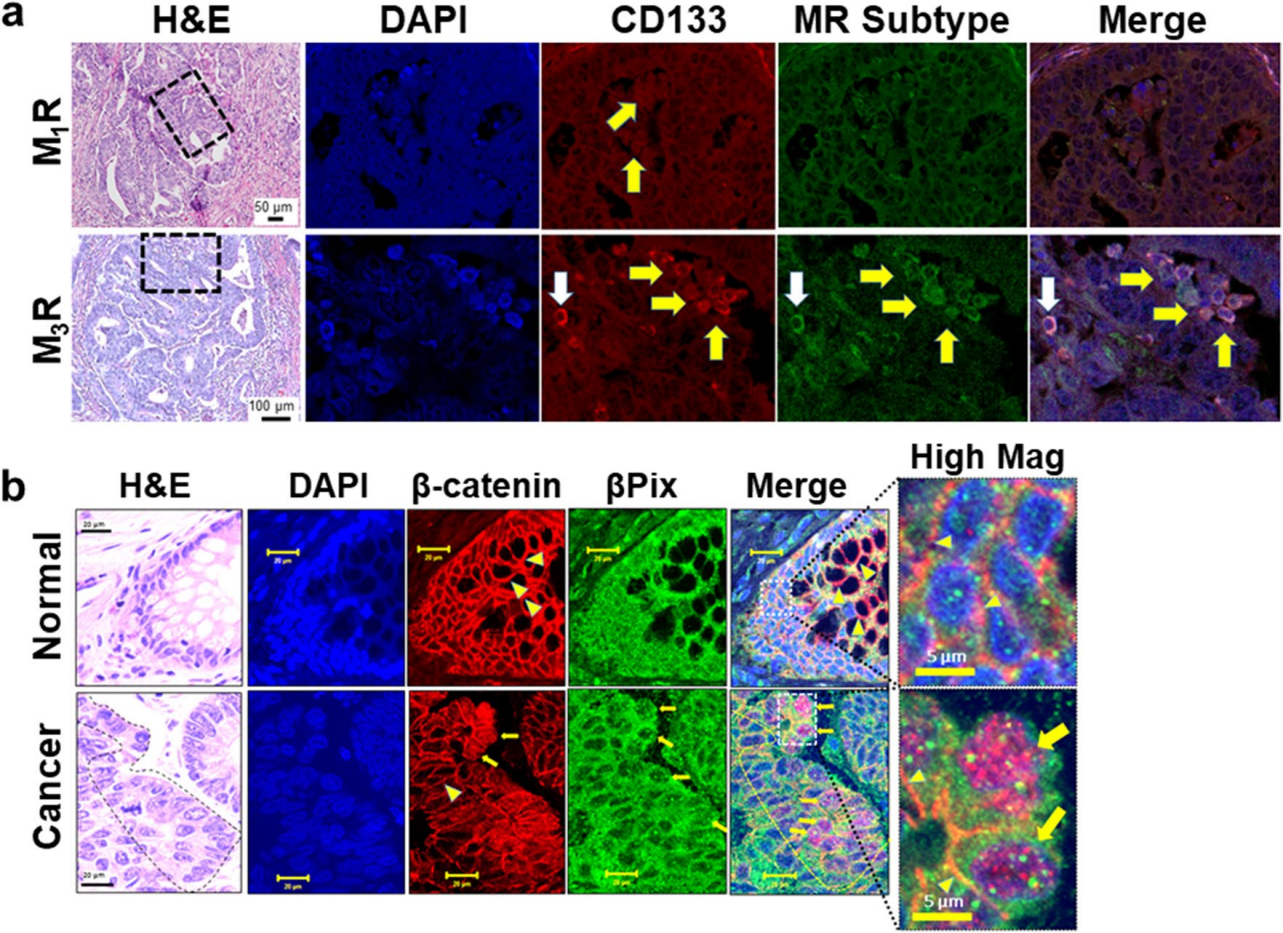
Colocalization of β-catenin and βPix in the nuclei of human colon cancer cells that overexpress M_3_R. (**a**) Overexpression of M_3_R, but not M_1_R, was observed in colon cancer stem cells. Images show H&E (left panels) and immunofluorescent staining with DAPI (nuclear stain, blue), CD133 (red-Alexa Fluor 594), M1R, and M3R (both green-Alexa Fluor 488), and merged images. Dashed boxes in H&Es show areas from which images were enlarged. CD133 staining reveals scattered colon cancer stem cells (white arrow) and colon cancer stem cell clusters around blood vessels (invasive front; yellow arrows). Top panels: M_1_R overexpression was not detected in colon cancer stem cells. Bottom panels: M_3_R overexpression was detected in colon cancer stem cells. Size bars in H&E images are 50 µm for M_1_R staining and 100 µm for M_3_R staining. (**b**) β-catenin and βPix colocalize in the nucleus of invasive human colon adenocarcinoma cells. Normal colon (top) and poorly differentiated cancer (bottom) tissues obtained at surgery from the same person were stained with H&E and DAPI, and immunostained with anti-β-catenin and anti-βPix antibodies. In normal colon and the cancer core, β-catenin and βPix staining was primarily membranous (arrowheads). In contrast, in cells at the invasive front (delineated by dashed lines in the H&E and merged images), β-catenin and βPix were co-localized to dysplastic nuclei (arrows). Size bars are 20 μm except for 5 μm in the high magnification (High Mag) images. Images are representative of n = 6 cancers.

Normal and neoplastic colon epithelial cells express a mix of M_1_R and M_3_R^39^. Thus, we initially explored differences in the relative expression and distribution of M_1_R and M_3_R in human colon cancer and compared this to the expression pattern of βPix (Fig. 1). Using anti-MR antibodies we^5,40^ and others^41^ previously used for IHC, we compared M_3_R to M_1_R expression in surgical specimens of sporadic colon cancer by immunohistochemistry (IHC). As recommended by Jositsch et al.^42^, we validated anti-M_1_R and anti-M_3_R antibody specificity using tissues from wild-type, M_1_R-, M_3_R-, and dual M_1_R/M_3_R-deficient mice (Supplemental Fig. 1a). We used these validated MR-selective antibodies to compare M_1_R and M_3_R expression in tissue samples from human normal colon epithelium (Supplemental Fig. 1b,c) and colon adenocarcinomas (Fig. 1). In sections from the normal human colon, we observed equivalent epithelial cell staining for M_1_R and M_3_R (Supplemental Fig. 1b,c). In contrast, at the tumor invasive front, CD133-positive colon cancer stem cells, clustered around blood vessels, demonstrated intense M_3_R but not M_1_R staining (Fig. 1a, top panels). The use of CD133, one of the first identified colon cancer stem markers, for this purpose has been validated by many investigators^43^. In the same areas demonstrating intense M_3_R staining, we detected nuclear co-localized βPix and β-catenin staining (Fig. 1b). Our findings that M_3_R but not M_1_R were overexpressed in colon cancer stem cells with colocalized overexpression of βPix and β-catenin are compatible with our hypothesis that M_3_R regulate the interaction between βPix and β-catenin, and hint at its functional importance.

### CHRM3/M_3_R and ARHGEF7/βPix are overexpressed in colon cancer

We examined the relationship between *CHRM3*/M_3_R and *ARHGEF7*/βPix expression in colon cancer. *CHRM3*/M_3_R is overexpressed in 60–80% of colon cancers^3–5^. Lei et al. reported *ARHGEF7*/βPix overexpression in colon cancer cell lines and in a small set of tissues; *ARHGEF7* expression levels were significantly increased in advanced stage colorectal cancer (TNM stage III) compared to early-stage disease (TNM stage I/II)^36^. Thus, *ARHGEF7*/βPix and *CHRM3*/M_3_R appear to share expression patterns in progressive colon neoplasia.

To confirm that *ARHGEF7* and *CHRM3* mRNA levels are increased in cancer compared to normal colon, we interrogated publicly available databases; Oncomine^44^, Gene Expression Profiling Interactive Analysis^45^, Human Protein Atlas ^46^, and the UALCAN server^47^. Per Oncomine, in 12 datasets *ARHGEF7* mRNA transcripts are overexpressed 2.640-fold in cancer compared to normal colon (*p* = 6.05E-12)^44,48^. *CHRM3* mRNA transcripts are overexpressed 2.411-fold in cancer compared to normal colon (*p* = 3.81E-15)^44,48^. *CHRM3* and *ARHGEF7* are in the top 10% overexpressed colon cancer genes^44,48^.

**Figure 2:**
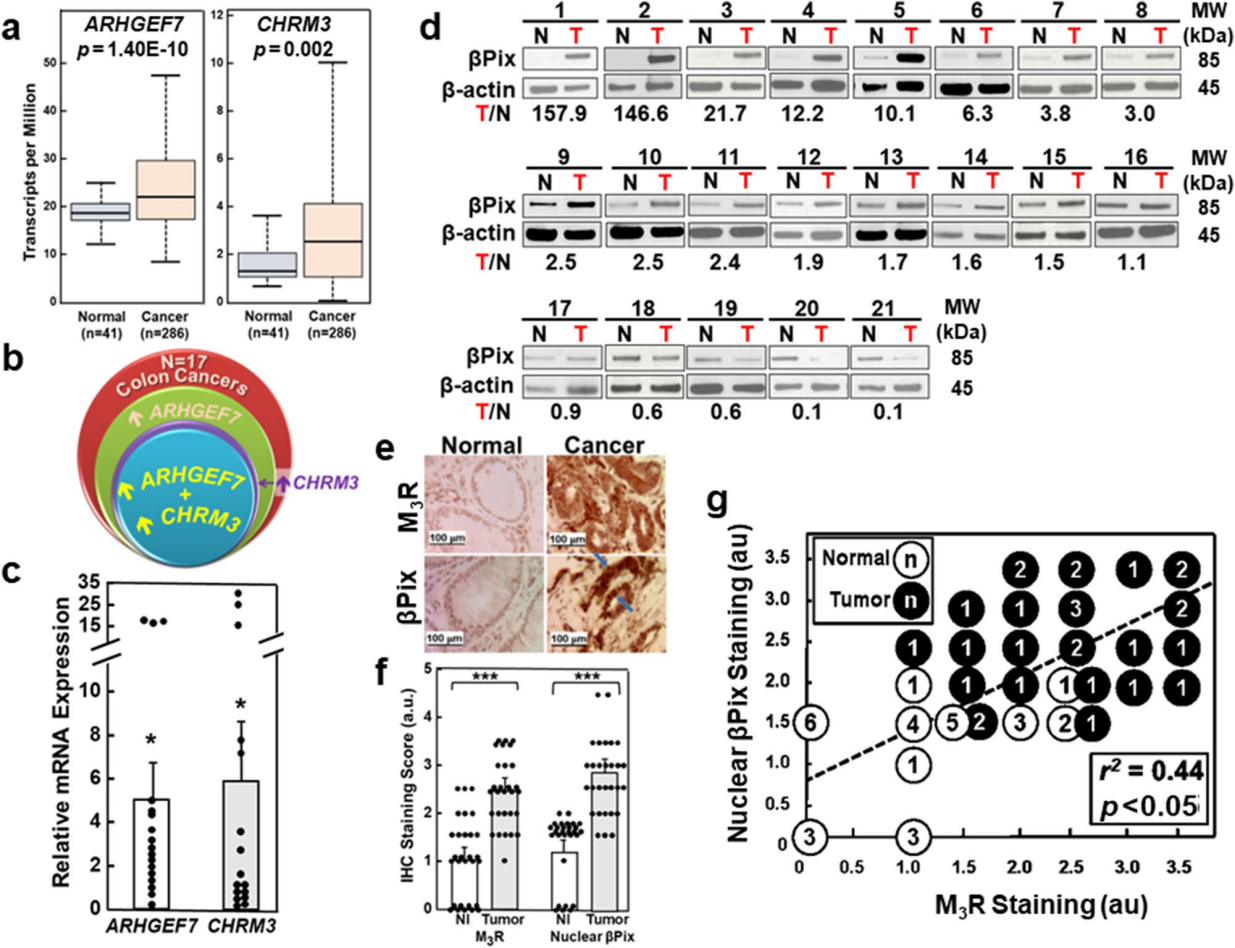
*ARHGEF7*/βPix and *CHRM3*/M3R overexpression in colon cancer is associated with nuclear βPix expression. (**a**) βPix (*ARHGEF7*) and M_3_R (*CHRM3*) overexpression in colorectal cancer. UALCAN server analysis^47^ reveals *ARHGEF7* (left panel) and *CHRM3* (right panel) mRNA transcripts are overexpressed in colon cancer (n = 286 patients) compared with normal colon (n = 41 patients). For normal colon, maximum, upper quartile, median, lower quartile, and minimum values, respectively, are 24.615, 20.3421, 18.244, 16.709, and 11.871 for *ARHGEF7* and 3.645, 2.055, 1.272, 1.069, and 0.678 for *CHRM3*. For colon cancer, these values, respectively, are 46.986, 29.116, 21.734, 17.029 and 8.098 for *ARHGEF7* and 10.027, 4.072, 2.528, 1.094, and 0.02 for *CHRM3*. (**b**) Patterns of *ARHGEF7* and *CHRM3* co-expression in colon cancer. mRNA was measured by qPCR in 17 colon cancers and adjacent normal colon tissue. *CHRM3* and *ARHGEF7* expression were increased greater than two-fold in 47% and 71% of cancers, respectively; both genes were co-overexpressed in 41% of cancers. (**c**) *ARHGEF7* and *CHRM3* gene expression are increased in colon cancer. *ARHGEF7* and *CHRM3* mRNA expression was significantly increased in cancer (bars represent means ± SE normalized to *β-2 microglobulin*; *p < 0.05 compared to adjacent normal colon). (**d**) βPix protein expression in colon cancer. βPix protein expression was increased up to 100-fold in 16 of 21 colon cancers (**T**) compared to adjacent normal colon (N). Immunoblots were arranged in order of decreasing βPix protein expression in tumors. Original uncut immunoblots are shown in Supplemental Materials. (**e**) Representative M_3_R and βPix immunostaining. Arrows show nuclear βPix staining. Size bars, 100 μm. (**f**) M_3_R and βPix staining was significantly increased in cancer compared to adjacent normal colon (***, *p* < 0.001). Bars, means ± SE of 29 samples. (**g**) Correlation between M_3_R and nuclear βPix immunostaining in normal colon (◯, N = 29) and cancer (⬤, N = 29) (numbers in symbols represent multiplicity of samples with same result); Pearson correlation coefficient = 0.44, *p* < 0.05. Dashed line = ‘best fit’. MW, molecular weight.

UALCAN analysis^47^ revealed *ARHGEF7* transcripts were tenfold more abundant in normal colon and cancer compared to *CHRM3* transcripts, consistent with generally low GPCR expression^49^. Compared to normal colon, median *ARHGEF7* and *CHRM3* transcript levels were higher in adenocarcinomas [21.734 vs. 18.244 per million for *ARHGEF7* (*p* = 1.3962E-10); 2.528 vs. 1.272 for *CHRM3* (*p* = 0.00236)] (Fig. 2a). Although UALCAN analysis failed to reveal a correlation between *ARHGEF7* mRNA transcript expression and survival, lower levels of *CHRM3* expression were associated with prolonged survival (*p* = 0.031; Supplemental Fig. 2a)^47^. *A*nalysis of the TCGA PanCancer Atlas dataset [333 colon, 137 rectal, 56 mucinous colorectal adenocarcinomas^50^] using cBioPortal (https://www.cbioportal.org) revealed frequent *ARHGEF7* amplification in colorectal cancer (Supplemental Fig. 2a). *ARHGEF7* and *CHRM3* mutations are uncommon (Supplemental Fig. 2c–f); primarily missense mutations were identified in 2.4 and 3%, respectively, of 526 specimens.

We compared relative *ARHGEF7* and *CHRM3* mRNA expression in 17 fresh human adenocarcinomas and paired normal colon. *ARHGEF7* and *CHRM3* were overexpressed two-fold or greater in eight (47%) and 12 (71%) cancers, respectively; *ARHGEF7* and *CHRM3* were both over-expressed in seven of 17 cancers (41%; Fig. 2b/c). Consistent with *ARHGEF7* overexpression, βPix protein was overexpressed in 16 of 21 cancers (76%; *p* = 0.0017, Fisher’s exact test; tumors 1–16 in Fig. 2d). In 11 samples (52%) normalized using β-Actin controls, βPix signal intensity was more than two-fold greater in tumors compared to paired normal colon.

We compared the subcellular distribution of βPix in 29 paired colon cancers and normal colon, and its correlation with M_3_R expression (Fig. 2e). IHC staining intensity was scored in 0.5 increments from 0 to 3.5 by a senior pathologist masked to the purpose of the analysis. Nuclear βPix staining was 2.5-fold greater in cancer. Likewise, M_3_R staining was increased in cancer (Fig. 2f). M_3_R expression correlated with nuclear βPix staining (*r*^*2*^ = 0.44; *p* < 0.05), and both βPix and M_3_R immunostaining were more intense in cancer compared to normal tissue (Fig. 2g).

### MR activation stimulates cytosolic and nuclear βPix binding to β-catenin

**Figure 3:**
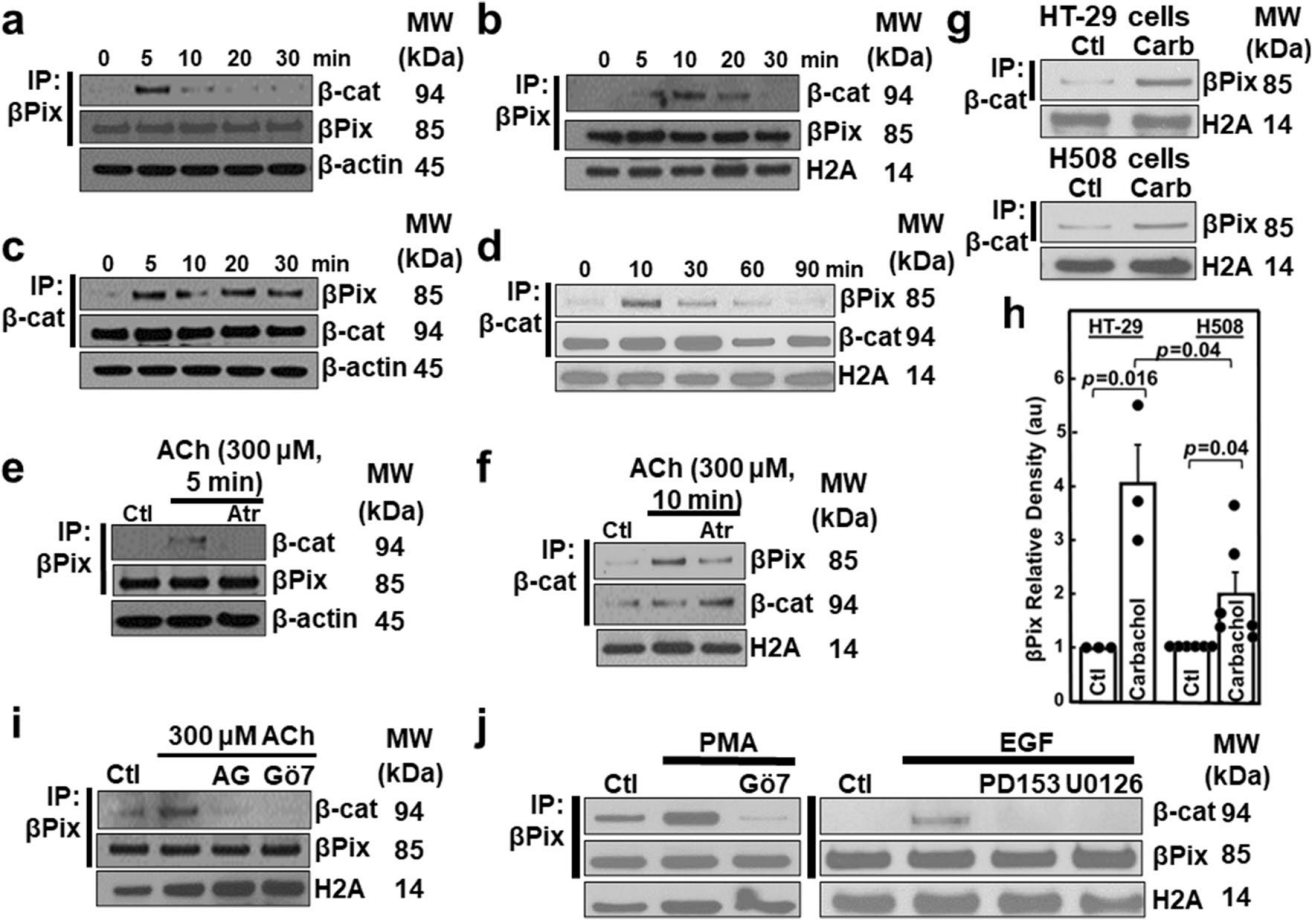
MR activation stimulates βPix binding to β-catenin. (**a-f**) At the times shown, HT-29 cells were treated with 300 μM ACh. Cytosolic (**a, c**) and nuclear (**b, d**) fractions were probed after IP. (**e–f**) Pre-incubating cells with atropine (Atr, 5 μM for 30 min) for the indicated times blocked ACh effects in both the cytosol (**e**) and the nucleus (**f**). Ctl, control; β-actin and histone 2A (H2A) were used as cytosolic and nuclear fraction loading controls. (**g**) HT-29 and H508 human colon cancer cells were treated with 100 µM carbamylcholine (carb). Nuclear fractions were probed for βPix after IP; histone 2A (H2A) is a loading control. (**h**) Relative density (au, arbitrary units) was measured in six different βPix immunoblots performed as illustrated in (**g**), normalized to the loading control, and expressed as a function of βPix expression in unstimulated cells. N = 3 and 6 experiments for HT-29 and H508 cells, respectively. Ctl, control. Histone 2A (H2A) was a nuclear loading control. (**i**) HT-29 cells were pre-incubated with inhibitors of EGFR (AG1478) and PKCα/β1 (Gö6976) for 60 min before adding 300 µM ACh for an additional 10 min. After treatment, nuclear fractions were immunoprecipitated with anti-βPix antibody followed by immunoblotting as indicated. Histone 2A (H2A) was a nuclear marker. (**j**) In the left panel, HT-29 cells were treated with 50 nM PMA for 10 min with or without pre-incubation for 45 min with a PKC inhibitor (5 μM Gö6976). In the right panel, HT-29 cells were incubated with 10 ng/ml EGF for 10 min with or without preincubation for 60 min with EGFR (5 µM PD153035) and MEK (10 µM U0126) inhibitors. Nuclear fractions were probed by immunoprecipitation with anti-βPix antibody and immunoblotting with anti-β-catenin and anti-βPix antibodies. MW, molecular weight. Original uncut immunoblots are shown in Supplemental Materials.

In established and primary human colon cancer cell cultures, we explored whether MR activation augmented βPix/β-catenin binding. In cytosolic and nuclear fractions from HT-29 and H508 cells that overexpress M_3_R and have mutated and wild-type *APC*, respectively^51^, we assessed binding of *endogenous* βPix to β-catenin. We used an antibody directed against the βPix SH3 domain to precipitate βPix followed by immunoblotting extracts with anti-β-catenin and anti-βPix antibodies (Fig. 3a/b) and reversed the sequence by immunoprecipitating β-catenin and immunoblotting for βPix and β-catenin (Fig. 3c/d). In acetylcholine (ACh)-treated HT-29 cells, we detected increased cytosolic binding of βPix to β-catenin (maximal by 5 min; Fig. 3a/c), followed by increased nuclear binding of βPix to β-catenin (maximal by 10 min; Fig. 3b/d). The actions of ACh were blocked by atropine (Fig. 3e/f). We duplicated these results in a second colon cancer cell line using a different MR agonist. Treating either HT-29 or H508 colon cancer cells with carbamylcholine (carbachol) induced two- to four-fold increased βPix binding to β-catenin (Fig. 3 g/h).

Post-M_3_R signal transduction is mediated by kinase-dependent mechanisms involving activation of PKC-α and/or transactivation of EGFR with downstream activation of the mitogen-activated protein kinase (MAPK)/ERK pathway; there is extensive crosstalk between signaling pathways^52^. To explore post-receptor signaling underlying MR agonist-induced nuclear binding of βPix to β-catenin, we used previously validated chemical inhibitors^52^. Pre-treating HT-29 cells with Gö6976, a PKC inhibitor, or with AG-1478, an EGFR inhibitor, nearly abolished MR agonist-induced binding of βPix to β-catenin (Fig. 3i). To confirm PKC and EGFR activation play a role in MR agonist-induced binding of βPix to β-catenin, we treated HT-29 colon cancer cells with phorbol 12-myristate, 13-acetate (PMA), a selective PKC activator and epidermal growth factor (EGF). PMA induced robust binding of *endogenous* βPix to β-catenin, an action inhibited by Gö6976 (Fig. 3j, left). EGF had a more modest effect on the binding of *endogenous* βPix to β-catenin (Fig. 3j, right). We concluded that post-MR signaling primarily via PKC, most likely PKC-α^52^, activation, but also via EGFR activation mediate MR agonist-induced binding of βPix to β-catenin.

**Figure 4:**
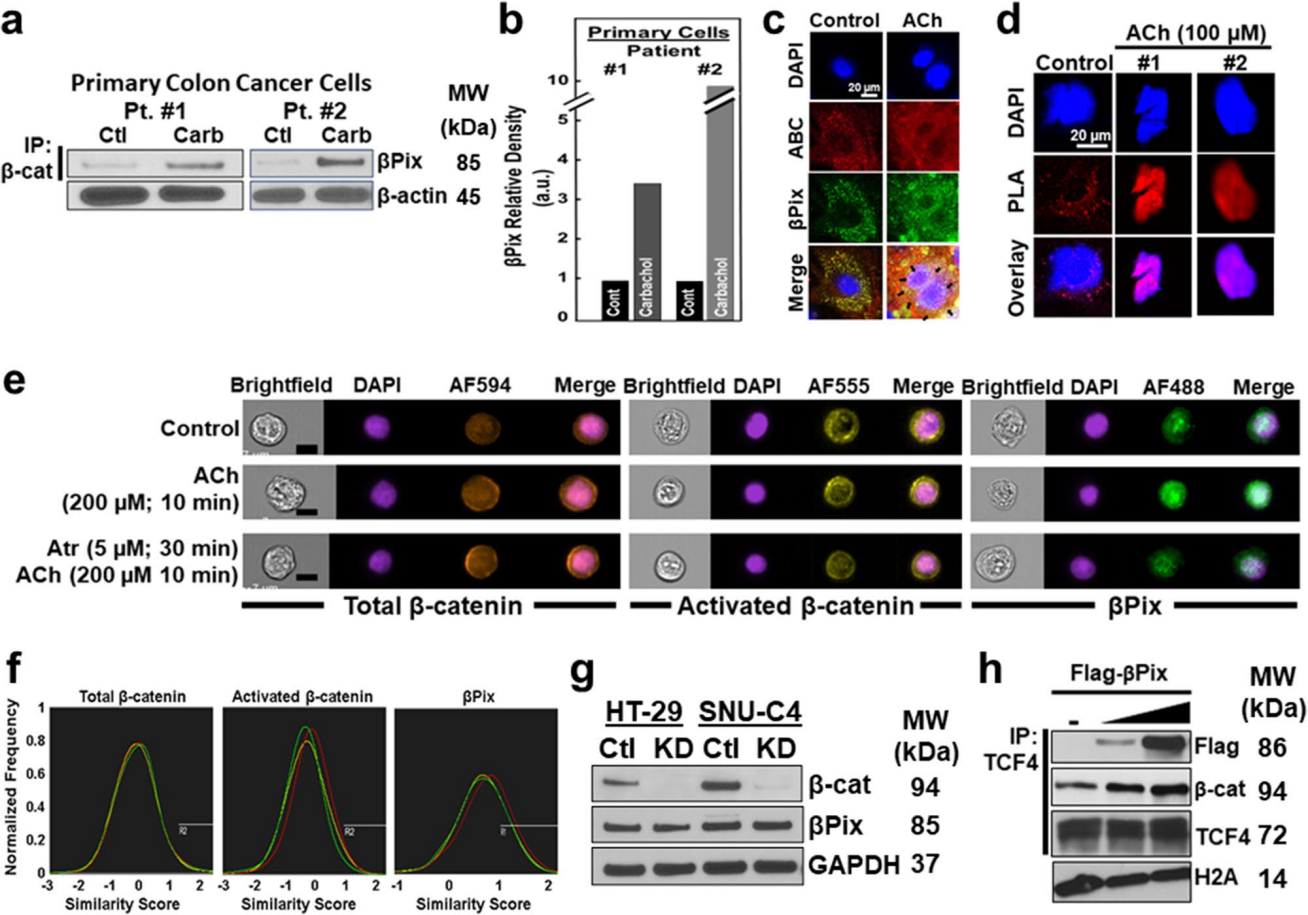
MR agonist treatment of colon cancer cells induces co-localized cytoplasmic and nuclear βPix and β-catenin. (**a**) MR agonist stimulates βPix binding to β-catenin in primary colon cancer cells. Cells were treated with 100 µM carbamylcholine (carbachol; 10 min, 37 °C). Cytosolic fractions were immunoprecipitated with anti-β-catenin antibody, then immunoblotted with anti-βPix antibody. β-actin was a loading control. (**b**) Relative βPix signal intensity in extracts from vehicle- and carbachol-treated primary colon cancer cells. au, arbitrary units. (**c**) MR agonist-induced βPix and β-catenin co-localization. Primary colon cancer cells were treated with vehicle or ACh (100 µM, 10 min), then stained with DAPI (*blue*), anti-activated β-catenin (ABC, *red*), and anti-βPix (*green*) antibodies. In the merged images, *yellow* in the cytoplasm and *purple* in the nucleus undergoing mitosis (arrows) reveal co-localized βPix and β-catenin. (**d**) Proximity ligand assay (PLA) reveals MR agonist-induced nuclear βPix/β-catenin co-localization. Images show DAPI- and PLA probe (*red*)-stained primary colon cancer cells. Left, PLA reveals co-localized βPix and β-catenin in the cytoplasm of untreated cell (control; red dots). In primary colon cancer cells (middle and right), ACh stimulated nuclear co-localization of βPix and β-catenin (*purple* in overlay). (**e**) Dual-color images are shown after using flow cytometry to view HT-29 cells stained with fluorescence-tagged antibodies and nuclear dye [nucleus (DAPI), total β-catenin (AF594), activated β-catenin (AF555), and βPix (AF488)]. βPix nuclear translocation triggered by ACh (200 µM, 10 min) was blocked by pretreating cells with atropine (5 µM, 30 min). Left to right: brightfield, nucleus (blue), and, respectively, total β-catenin (brown), activated β-catenin (yellow), βPix (green), and merged images are shown. Scale bars, 10 µm. (**f**) Fluorescent cell sorting. Similarity scores for stained HT-29 cells are shown [control, green; ACh treatment, red; atropine pre-treatment then ACh, yellow]. (**g**) β-catenin knockdown does not alter βPix expression. HT-29 and SNU-C4 cells were transfected with β-catenin siRNA. Extracts were immunoblotted for β-catenin, βPix, and GAPDH (used as a loading control). Results shown are representative of three experiments. (**h**) βPix overexpression enhances nuclear β-catenin binding to TCF4. HCT116 cells were transfected with Flag-βPix and nuclear extracts were immunoprecipitated with anti-TCF4 antibody and immunoblotted for Flag and β-catenin. Histone 2A (H2A) was used as a loading control. MW, molecular weight. Original uncut immunoblots for (**a**, **g**), and h are shown in Supplemental Materials.

To validate these findings, we used different experimental approaches with cells from fresh primary adenocarcinomas and established cell lines. In cytosolic extracts from primary colon cancer cells treated with carbachol we detected robust βPix binding to β-catenin (Fig. 4a). Quantitative measurements of cytosolic fractions revealed that carbachol stimulated three- to tenfold increases in anti-βPix antibody band density (Fig. 4b), supporting the clinical relevance of our findings with established cell lines. Our ability to detect nuclear βPix binding to β-catenin in primary cells was compromised by sluggish in vitro cell proliferation and cell-to-cell variation. To circumvent technical difficulties in assessing MR agonist-induced nuclear binding of βPix to β-catenin in primary colon cancer cells, we employed immunofluorescence (IF) microscopy, proximity ligation assays (PLA)^53^, and a novel approach combining fluorescence microscopy with flow cytometry^54^.

Using primary colon cancer cells and IF microscopy (Fig. 4c, left), we detected modest *constitutive* cytoplasmic colocalization of βPix and activated β-catenin (ABC), using an antibody that recognizes activated β-catenin unphosphorylated at Ser-37 and Thr-41 residues^55^. Treating primary colon cancer cells with ACh stimulated striking cytoplasmic and nuclear colocalization of βPix and activated β-catenin (Fig. 4c, right; arrows indicate nuclear co-localized βPix and activated β-catenin).

We used PLA which detects proteins colocalized within 40 nm ^53^, as another test of MR agonist-induced nuclear co-localization of βPix and β-catenin. Applying PLA to two fresh colon cancer preparations, we identified robust MR agonist-induced nuclear signals consistent with colocalized βPix and β-catenin (representative nuclei shown in Fig. 4d), actions blocked by atropine (not shown).

The 5-min time lag between MR agonist-induced βPix binding to β-catenin in the cytoplasm and the nucleus could represent delayed nuclear translocation of βPix, β-catenin, or the βPix/β-catenin complex. Alternatively, it could result from delayed nuclear translocation of signaling molecules (e.g., PKCα). We used fluorescence cell sorting to explore if MR activation stimulated nuclear translocation of βPix or β-catenin. After treating HT29 cells with 200 µM ACh for 10 min, we measured shifts in nuclear localization of total β-catenin, activated β-catenin, and βPix. We failed to detect an increased nuclear signal for either total or activated β-catenin (Fig. 4e, left and middle). In contrast, in ACh-treated cells we observed a modest nuclear signal for βPix that was abolished by atropine (Fig. 4e, right). To assess overall changes in nuclear localization, we used the ‘similarity score’ to measure the change in similarity between protein and nuclear images (Fig. 4f). Post MR agonist treatment, median similarity scores showed no significant changes in nuclear localization of total or activated β-catenin (Fig. 4f, left and middle panels; Table 1). As shown in Fig. 4f (right), compared to control, there was a rightward shift in βPix similarity scores, abolished by pretreating cells with atropine. Nonetheless, these changes representing only a 4% change in nuclear signal intensity were unlikely to be biologically meaningful and did not achieve statistical significance (Table 1).

**Table 1:** Similarity Scores reveal no MR agonist-induced nuclear translocation of total and activated β-catenin and βPix.

Treatment	Similarity scores					
	Total β-catenin		Activated β-catenin		βPix	
	Mean	SD	Mean	SD	Mean	SD
Control	−0.38	0.73	−0.36	0.66	0.67	0.48
ACh	−0.35	0.72	−0.11	0.64	0.76	0.47
Atr + ACh	−0.40	0.71	−0.26	0.69	0.69	0.47

SD, standard deviation, ACh, acetylcholine, Atr, atropine.

We were curious to see if β-catenin transcriptional activity modulated βPix expression. We verified that siRNA-treated HT-29 and SNU-C4 cells had reduced β-catenin expression; Fig. 4g, top panel, shows a negligible signal for β-catenin expression in cells transfected with β-catenin siRNA. β-catenin knockdown did not alter βPix expression (Fig. 4g).

Collectively, our findings suggest M_3_R agonists stimulate binding of pre-existing nuclear βPix to β-catenin and not cytoplasm-to-nucleus translocation of either βPix or β-catenin. Thus, our working model suggests M_3_R activation stimulates βPix binding to β-catenin independently within the cytoplasm and nucleus. We performed a pilot experiment to test whether nuclear βPix bound to β-catenin could participate as a TCF-4 transcriptional cofactor. We used anti-TCF-4 antibodies to determine whether *exogenous* Flag-tagged βPix augmented β-catenin binding to TCF-4. Immunoprecipitation assays using nuclear extracts derived from HCT116 colon cancer cells incubated with increasing concentrations of Flag-βPix revealed progressively augmented Flag-βPix and β-catenin binding to TCF-4 (Fig. 4h). These results suggest a mechanism whereby MR-stimulated nuclear binding of βPix to β-catenin can alter gene expression and cell function.

### βPix deficiency attenuates MR agonist-induced changes in cell function

Lei et al. reported that up- and down-regulating βPix expression in HCT116 and Lovo human colon cancer cell lines, respectively augmented and attenuated cell migration and invasion^36^. MR activation also stimulates cell migration^6^ and invasion^7,52^. Hence, we next examined whether transient and stable βPix deficiency alters MR agonist-induced cell proliferation, migration, and invasion.

**Figure 5:**
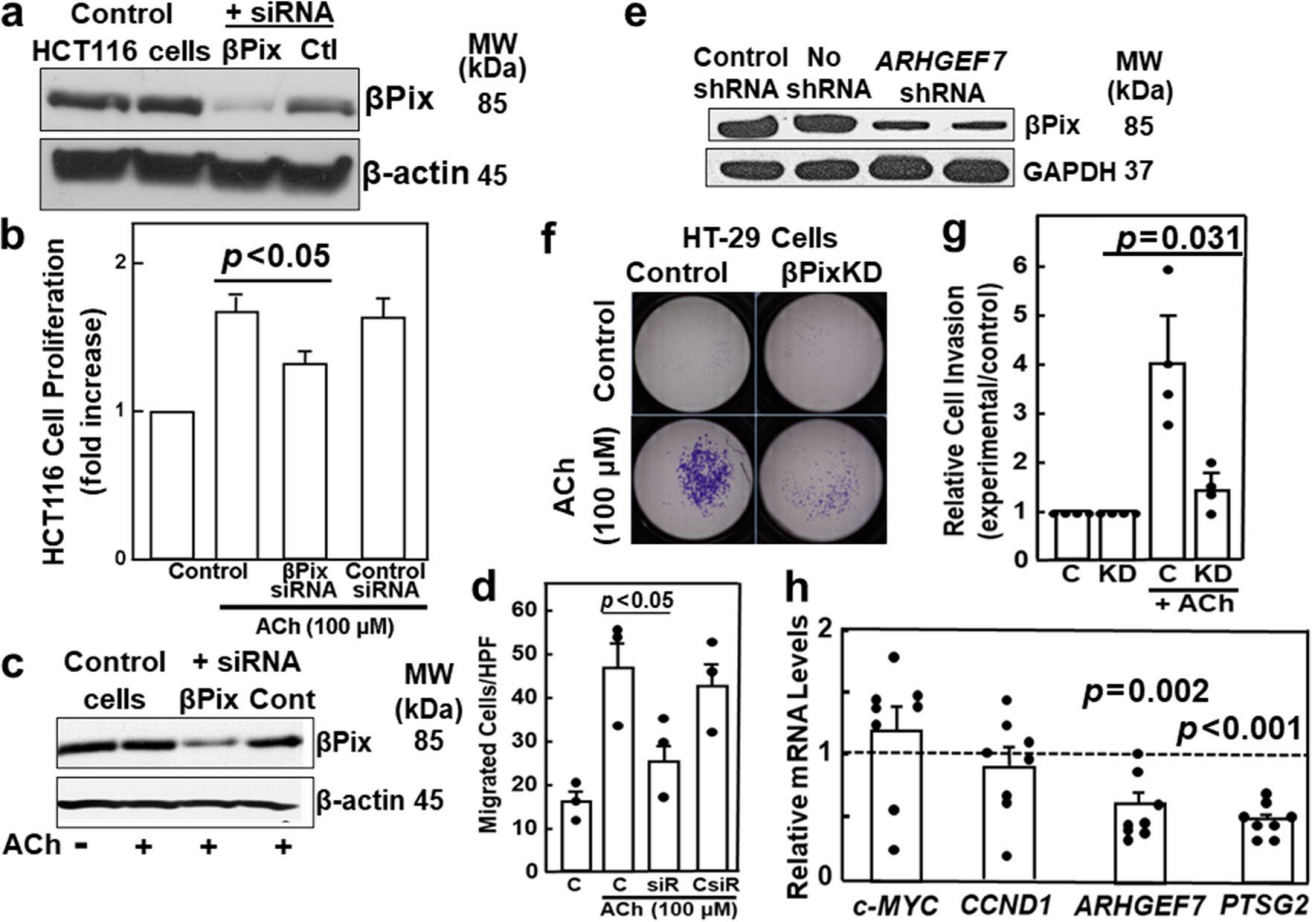
βPix deficiency attenuates MR agonist-induced changes in colon cancer cell function. (**a**) Extracts from control HCT116 cells and cells transfected with siRNA directed against *ARHGEF7* or control siRNA were immunoblotted for βPix. βPix expression was reduced by siRNA directed against *ARHGEF7* but not altered by transfection with control siRNA. β-actin was a loading control. (**b**) βPix contributes to MR agonist-induced colon cancer cell proliferation. Control HCT116 cells and cells transfected with 50 nM siRNA directed against *ARHGEF7* or 50 nM control siRNA were stimulated with 100 μM ACh for 48 h. Cell proliferation was measured using the CellTiter-Glo assay. Results are means of at least three independent experiments in triplicate. *, *p* < 0.05. (**c**) Extracts from control HT-29 cells and HT-29 cells transfected with siRNA directed against *ARHGEF7* or control siRNA were immunoblotted for βPix. βPix expression was reduced by *ARHGEF7* siRNA but not altered by treatment with 100 μM acetylcholine (ACh) for 24 h or transfection with control siRNA. β-actin was a loading control. (**d**) βPix knockdown attenuates colon cancer cell migration. HT-29 cells, placed in the upper chamber of uncoated inserts, were stimulated with 100 μM ACh for 24 h. Cells migrating to the underside of inserts were fixed, stained, and counted using light microscopy. Bars represent mean ± SE from three individual experiments. Symbols represent results from individual experiments (n = 3 per condition). (**e**) Immunoblotting confirms βPix protein expression was reduced by transfecting HT-29 cells with shRNA directed against *ARHGEF7*. GAPDH was a loading control. (**f**) βPix knockdown attenuates ACh-induced human colon cancer cell invasion. HT-29 cells were placed in the upper chamber of Matrigel-coated inserts and stimulated with vehicle or 100 μM ACh for 48 h. Cells invading to the underside of inserts were fixed, stained, and counted using light microscopy. (**g**) βPix knockdown attenuates ACh-induced colon cancer cell invasion. Bars represent mean ± SE from four individual experiments. Symbols represent individual experiments (n = 4 per condition). (**h**) shRNA knockdown of *ARHGEF7* expression reduced *PTGS2* mRNA levels *p* < 0.001). mRNA levels were measured by qPCR and normalized to *β-2 macroglobulin*. MW, molecular weight. Original uncut immunoblots for (**a**, **c**), and e are shown in Supplemental Materials.

Transfecting HCT116 cells with siRNA against *ARHGEF7* substantially reduced βPix expression (Fig. 5a). Transfecting cells with siRNA against *ARHGEF7*, but not control siRNA, reduced ACh-induced HCT116 cell proliferation (Fig. 5b; *p* < 0.05). We used the same approach to knock down βPix expression in HT-29 cells and confirmed ACh (100 μM for 24 h) did not alter βPix expression in naïve or transfected cells (Fig. 5c). ACh stimulated a three-fold increase in cell migration in control and mock transfected cells (Fig. 5d). ACh-induced cell migration was reduced more than 70% in βPix-deficient cells (Fig. 5d;* p* < 0.05). Next, we examined the effects of stable knockdown of βPix expression on cell invasion. shRNA against *ARHGEF7* robustly knocked down βPix expression in HT-29 cells; control shRNA had no effect (Fig. 5e). Whereas we detected limited Matrigel invasion by untreated control and βPix-deficient HT-29 cells, ACh stimulated a four-fold increase in cell invasion (Fig. 5f) that was reduced ~ 85% by βPix-deficiency (*p* = 0.031; Fig. 5g). Thus, βPix deficiency profoundly impaired MR agonist-induced colon cancer cell proliferation, migration, and invasion.

To gain additional mechanistic insights, we examined the effects of βPix deficiency on several genes associated with colon cancer progression; *c-MYC*, *CCND1*, and *PTGS2*, which encode c-Myc, β-catenin, and cyclooxygenase-2. In HT-29 colon cancer cells, βPix deficiency greatly reduced expression of *PTGS2* mRNA (*p* < 0.001; Fig. 5h), whose gene product, prostaglandin-endoperoxide synthase-2 [PTGS2; cyclooxygenase-2 (COX2)], catalyzes arachidonic acid metabolism.

### Intestinal βPix deficiency attenuates colon neoplasia in mice

**Figure 6:**
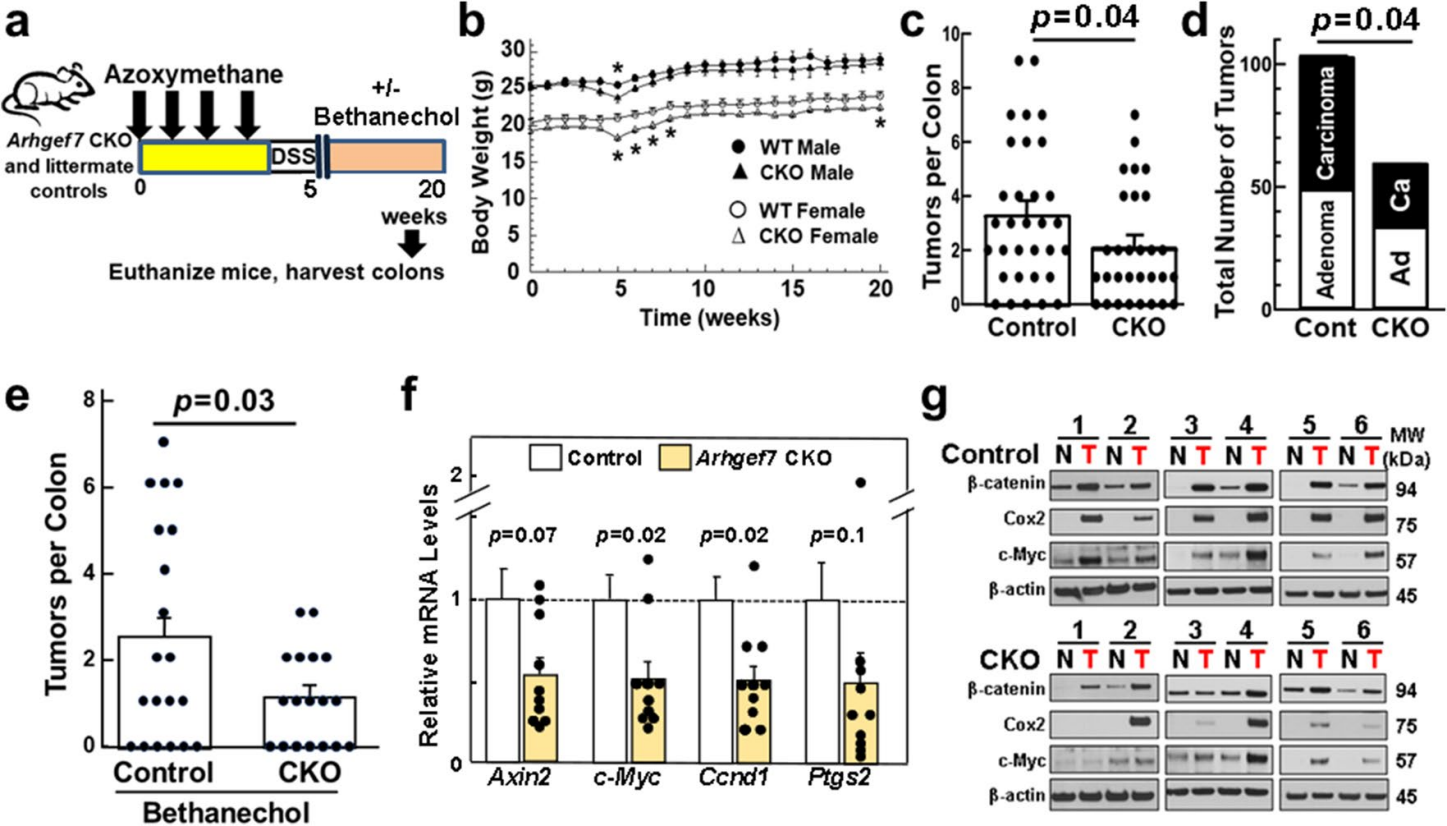
Colon neoplasia is attenuated in βPix-deficient mice. (**a**) Treatment scheme for azoxymethane (AOM)/dextran sodium sulfate (DSS)-induced colon neoplasia in mice. Colon neoplasia was induced with four weekly intraperitoneal injections of 7.5 mg AOM/kg mouse body weight. One week after the last dose of AOM, 2.5% DSS was added to the animals’ drinking water for five days. In some experiments, bethanechol (400 µg) was added to the animals’ drinking water for 15 weeks after AOM/DSS treatment. Mice were euthanized 20 weeks after the first AOM injection and their colons were harvested for analysis. (**b**) Mouse body weights over the 20-week treatment period. *Arhgef7* CKO and littermate control mouse weights were similar, including an approximately four-week period of weight loss associated with DSS treatment. Regardless of genotype, female mice weighed approximately 20% less than male mice. (**c**) Colon tumor numbers were reduced in *Arhgef7* CKO mice. CKO mice (n = 30) had fewer colon tumors than control mice (n = 32) (*p* = 0.04). Each symbol represents the number of colon tumors detected in one mouse. Bars represent means ± SE. (**d**) Reduced numbers of adenocarcinomas in AOM/DSS-treated *Arhgef7* CKO mice. Bar graph shows 24% fewer adenomas and 55% fewer adenocarcinomas in CKO compared to control mice. (*p* = 0.04). (**e**) Colon tumor numbers were reduced in *Arhgef7* CKO mice treated with a MR agonist. After AOM/DSS treatment, bethanechol (400 µg/ml) was added to the animals’ drinking water for 15 weeks. CKO mice (n = 18) had substantially fewer colon tumors than littermate control mice (n = 20) (*p* = 0.03). Each symbol represents the number of colon tumors detected in one mouse. Bars represent means ± SE. (**f**) Expression of colon cancer-related genes was measured in adenocarcinomas harvested from 10 AOM/DSS-treated *Arhgef7* CKO and 14 littermate control mice. Each symbol represents the mRNA level in an adenocarcinoma from one mouse relative to the mean values for that gene in control mice (arbitrarily set at 1). Bars represent means ± SE. (**g**) Representative immunoblots show paired expression of colon cancer-related proteins in normal colon and colon adenocarcinomas harvested from AOM/DSS-treated littermate control (n = 6) and *Arhgef7* CKO (n = 6) mice. Original uncut immunoblots are shown in Supplemental Materials.

We explored the role of βPix in vivo by treating 30 (15 male and 15 female) *Arhgef7* CKO mice and 32 (15 male and 17 female) littermate controls with AOM/dextran sodium sulfate (DSS) and harvesting colons after 20 weeks (Fig. 6a). Body weights were similar in CKO and control mice; mice lost weight during DSS treatment (Fig. 6b). *Arhgef7* CKO mice had 39% fewer colon tumors (3.25 ± 0.47 tumors/control versus 2.00 ± 0.37 tumors/CKO mouse) (Fig. 6c) − 55% fewer adenocarcinomas and 24% fewer adenomas (both *p* = 0.04; Fig. 6d). In a subsequent experiment, we added bethanechol, a non-subtype selective MR agonist, to the drinking water of 18 *Arhgef7* CKO and 20 control mice for the 15 weeks after AOM/DSS treatment (Fig. 6a). *Arhgef7* CKO mice had 58% fewer colon tumors than control mice (Fig. 6e; *p* = 0.03), a stronger effect of βPix deficiency than without MR agonist treatment (*p* = 0.02, Fisher’s exact test).

Having previously observed a relationship between MR activation and expression of *cMyc*, *Ccnd1*, and *Ptgs2* (*Cox2*)^56^, we compared expression of these genes in adenocarcinomas from CKO and control mice. mRNA expression levels for *cMyc* and *Ccnd1*, the genes encoding c-myc and cyclin D1 were reduced ~ 50% in tumors from *Arhgef7* CKO mice (Fig. 6f; *p* = 0.02). Removing an ‘outlier’ from the *Ptgs2* dataset (the highest value, 2.05, was > 2 SD from the mean), uncovered 70% reduced *Ptgs2* expression in βPix-deficient cancers (0.32 ± 0.08, mean ± SE; *p* = 0.03).

We next examined c-Myc, β-catenin, and Cox2 protein expression in normal colon and tumors harvested from six *Arhgef7* CKO and six control mice. Whereas β-actin loading controls were similar in gels from control and CKO mice, except for Cox2 signals in CKO mice 2 and 4, expression of colon cancer-related proteins was substantially reduced in βPix-deficient adenocarcinomas (Fig. 6g). CKO mouse number 2 was the same mouse with the ‘outlier’ *Ptgs2* mRNA signal (Fig. 6f). We confirmed mouse genotypes, so we cannot presently explain how mice 2 and 4 escaped downregulated Cox2 expression observed with βPix deficiency in other mice.

Lastly, we sought an association between *ARHGEF7* and *PTGS2* expression in colon cancer. Using methods described previously^57^, we interrogated public gene databases for paired normal colon and cancer (n = 78 for both). We failed to detect a correlation between *ARHGEF7* and *PTGS2* mRNA expression (Supplemental Fig. 3a). Likewise, we failed to detect a correlation between *PTGS2* and *CHRM3* mRNA expression (Supplemental Fig. 3b).

## Discussion

There is growing interest in noncanonical roles for GTP-binding proteins that modulate post-receptor signaling. Such interactions fine-tune signaling that might result in unbridled cell proliferation and other attributes of neoplasia. *ARHGEF6* and *ARHGEF7,* encode two proteins, αPix and βPix, respectively, that interact functionally with many molecules [reviewed in^58^]. Of more than 40 ‘proven’ βPix binding interactions with oligomeric protein partners [Table S1 in^58^], complexes between βPix and the Src family of protein tyrosine kinases, GPCR-kinase-interacting proteins, EGFR, and β-catenin are likely most relevant to colorectal neoplasia. Yet, despite the potential biological and pathological consequences of βPix-binding interactions, little was known regarding how these interactions are regulated.

Previously, we uncovered a noncanonical role for βPix, wherein its binding to β-catenin regulates transcriptional activity and cell function^38^. Here, we provide evidence that M_3_R activation regulates this interaction. In addition to demonstrating that *CHRM3*/M_3_R and *ARHGEF7*/βPix are coordinately overexpressed in colon cancer, immunoprecipitation experiments revealed that in the cytosol and nucleus M_3_R activation provokes time-dependent βPix binding to β-catenin by a PKC-dependent mechanism. Crucially, the validify of these findings were strengthened by multiple lines of evidence from IF microscopy, PLA, and fluorescence cell sorting assays. Although the data shown in Fig. 1 suggest nuclear colocalization of βPix and β-catenin is exaggerated in colon cancer stem cells, the results of experiments shown in Fig. 4c,d,e and g, clearly demonstrate this also occurs in the general population of colon cancer cells − it is not limited to colon cancer stem cells. Pilot data suggested nuclear βPix may be a cofactor in the β-catenin/TCF4 transcription factor complex, thereby regulating β-catenin target gene expression. Supporting the functional importance of MR agonist-induced βPix binding to β-catenin, we found that reducing βPix expression in vitro attenuated M_3_R agonist-induced colon cancer cell proliferation, migration, and invasion. Moreover, in the AOM/DSS murine model of colon cancer, mice with conditional intestinal epithelial cell βPix deficiency^59^ had significantly fewer colon tumors, primarily fewer adenocarcinomas, a finding exaggerated in animals treated with a MR agonist.

In human colon cancer cells, we observed that βPix knockdown reduced *PTGS2* expression. The expression of *PTGS2*, whose protein product, cyclooxygenase-2 (COX2), catalyzes the metabolism of arachidonic acid to prostaglandins and other bioactive molecules, must be carefully regulated. Elevated levels of COX2 are associated with the development and progression of colon neoplasia^60–62^ and reduced survival^63^; in both animal models and humans, inhibiting COX-2 activity with non-steroid anti-inflammatory drugs attenuates colon neoplasia. MR activation in human colon cancer cells up-regulates *COX2* expression^4^. We found *Ptgs2* expression was also greatly reduced in tumors from *Arhgef7* CKO mice with intestinal epithelial cell-selective βPix deficiency. Finding a connection between the M_3_R/βPix/β-catenin and *COX2* expression provides a mechanistic framework that may be clinically relevant. Indeed, the lack of a relationship between *ARHGEF7* and *PTGS2* expression in colon cancer suggests M_3_R activation is pivotal to augmenting *COX2* expression by a βPix-dependent mechanism.

Based on the current findings, we propose a model wherein post-MR signal transduction, mediated primarily by PKC-α activation, stimulates βPix binding to β-catenin in the cytoplasm and nucleus. Our preliminary data suggest nuclear βPix binds β-catenin/TCF4 transcription complexes, augmenting key β-catenin target gene expression, e.g., *PTGS2*, important for cancer progression^60–62^. Reduced βPix expression attenuates β-catenin target gene expression, cell function, and murine colon tumor formation. Nonetheless, we acknowledge that our work has limitations. In vitro cell models may not recapitulate complex in vivo interactions and murine cancer models, while mimicking key features of human colon cancer progression, do not fully mirror the development and progression of human colon neoplasia. Lastly, a putative role for βPix as a cofactor in the β-catenin/TCF4 transcription factor complex must remain speculative until confirmed by complementary approaches. Pursuing this intriguing finding and confirming a functional interaction between *endogenous* βPix and the β-catenin/TCF-4 transcription factor complex that alters β-catenin target gene expression requires luciferase reporter, electrophoretic mobility shift, chromatin immunoprecipitation, and other approaches beyond the scope of the current project and will be pursued in future work.

In conclusion, our findings identify a noncanonical role of βPix as an intermediary between M_3_R and β-catenin signaling that modulates colon cancer gene expression and cell function. Employing rigorous in vitro and in vivo experimental designs to test established human colon cancer cell lines and fresh human colon cancer cells using complementary innovative approaches, we uniformly observed M_3_R agonist-stimulated βPix/β-catenin binding in both the cytosolic and nuclear compartments and consistent effects on downstream expression of β-catenin target genes. Targeting the M_3_R/βPix/β-catenin axis has therapeutic potential. Our experience using *Arhgef7* CKO mice with intestinal epithelial cell-selective βPix deficiency suggests targeting intestinal βPix would be safe; *Arhgef7* CKO mice are healthy and fertile with modest phenotypic changes^59^. Lastly, since other upstream receptors act through PKC-α, our findings may be mechanistically relevant to other receptors and signaling pathways in colon neoplasia and other cancers in which muscarinic receptor signaling plays a prominent role.

## Materials and methods

### Chemicals and antibodies

Chemicals and reagents were obtained from the following sources: Matrigel from Corning (#356,231), azoxymethane (AOM) from MRIGlobal (#0061); and dextran sodium sulfate (DSS) from MP Biomedical (#160,110). If not otherwise specified, chemicals and reagents were from Sigma-Aldrich, St. Louis, MO, USA. Antibodies were obtained and used as follows: anti-βPix antibodies from Millipore (#07–1450-I; used for immunoblotting), Santa Cruz (#sc-393184; used for immunohistochemistry in murine tissue), and Sigma-Aldrich (#HPA004744; used for immunohistochemistry in human tissue); anti-total β-catenin from Abcam (#ab19381); anti-activated-β-catenin from Millipore (05–665-AF555); Alexa Fluor 488 from Invitrogen (#A21206); Alexa Fluor 594 from Invitrogen (#A21203); anti-GAPDH from Cell Signaling (#2118); anti-FLAG antibody from Sigma-Aldrich (#F1365); and anti-Ki67 from Bethyl Labs (#IHC-00375). PCR primers are shown in Supplemental Table 1.

### Animals

We created *Arhgef7*^*flox/flox*^-/Tg(Villin-Cre) mice (*Arhgef7* CKO mice) with intestinal epithelial βPix deficiency^59^. Mice were bred, housed, and treated under identical conditions in a pathogen-free room. To achieve microbiome equivalence, we co-housed animals throughout these studies until they were euthanized. Experiments were approved by the Institutional Animal Care and Use Committee of the University of Maryland, Baltimore, and VA Maryland Health Care System Research and Development Committee. All experiments were performed in accordance with institutional guidelines and regulations and the authors complied with the ARRIVE guidelines.

### Established human colon cancer cell lines

HT-29, H508, SNU-C4, and HCT116 human colon cancer cell lines were authenticated and purchased from American Type Culture Collection. HT-29 and HCT116 cells were grown in McCoy's 5A medium and H508 and SNU-C4 wells were grown in RPMI 1640 (Life Technologies-Thermo Fisher), both supplemented with 10% FBS. Cells were grown at 37ºC, with 5% CO_2_ in a humidified incubator and passaged weekly at subconfluence after trypsinization.

### Preparation and culture of primary human colon cells

We collected deidentified discarded fresh tissue samples from colon adenocarcinomas and adjacent normal colon from patients undergoing primary colon cancer resection at the University of Maryland Medical Center (Institutional Review Board exemption HP-00085101, 3/20/2019; no organs/tissues were obtained from prisoners). As described previously^64^, after washing (HBSS), we cut tissues into 1–2 mm^3^ sections, and placed them in 0.1% trypsin at 4 °C overnight. We then placed tissues in complete growth media (RPMI 1640 supplemented with 10% FBS with an antibiotic–antimycotic) and incubated the material at 37 °C for 15 min. We then washed tissues (HBSS) before additional incubation in 0.1% collagenase for 45 min at 37 °C (5% CO_2_). After filtration (40-μm filters), we centrifuged cells at 150 × g for 5 min at room temperature, resuspended cells in culture media, and seeded cells in T25 flasks.

### Proximity ligation assay (PLA)

We used the Duo-link In Situ-Fluorescence kit following both the manufacturer's instructions (Sigma-Aldrich) and published methods^65^. Briefly, we grew fresh primary cancers on slides, fixed in cold MeOH for 10 min and permeabilized with 0.1% Triton-X-100 for 10 min on ice. We incubated cells with blocking solution for 60 min and incubated overnight (4 °C) with anti-βPix and -β-catenin antibodies. We then incubated cells with PLA probes for 1 h, with ligation solution for 30 min, and amplification solution for 100 min (all at 37 °C). To identify cell nuclei, we stained slides with 4',6-diamidino-2-phenylindole (DAPI). We acquired images using an LSM 510 inverted confocal microscope; dots in the cell nucleus represented βPix molecules within 40 nm of β-catenin molecules.

### Quantitative RT-PCR (qPCR)

As described previously^59^, we synthesized first-strand cDNAs from 5 µg RNA (Superscript III First Strand Synthesis System for RT-PCR, Invitrogen) and performed qPCR using 50 ng cDNA, the SYBR Green PCR Master Mix (Applied Biosystems), and 0.5 μM in 20 μl forward and reverse primers. We designed primers (Supplemental Table 1) to span introns using the National Center for Biotechnology Information nucleotide database SIM-4 gene alignment program and on-line software (www.genscript.com/ssl-bin/app/primer). We performed qPCR using Step One (Applied Biosystems) with Power SYBR Green Master Mix (ABI). PCR conditions were as follows: 5 min at 95 °C followed by 37 cycles of 95 °C for 15 s, 60 °C for 20 s, 72 °C for 40 s, and final cycles at 95 °C for 15 s, 60 °C for 15 s, and 95 °C for 15 s. We normalized gene expression to *β*_*2*_*-microglobulin* (*B2M*) and analyzed qPCR data using the comparative *C*_T_ (2^–ΔΔ*C*^_T_) method.

### Immunoblotting and immunoprecipitation

For immunoblotting, we treated cells with MR agonists and antagonists at the concentrations and times indicated and, as described previously^38^, lysed cells in 20 mM Tris, pH 7.5, 100 mM NaCl, 5 mM MgCl_2_, 1 mM EDTA, 1% Triton X-100, 1 mM sodium fluoride, 1 mM sodium vanadate, 1 mM phenylmethylsulfonyl fluoride, 1 µg/ml pepstatin, and 1 µg/ml leupeptin. We separated equal amounts of protein by 7.5% sodium dodecyl sulfate–polyacrylamide gel electrophoresis, transferred onto polyvinylidene difluoride membranes (Millipore), immunoblotted, and visualized bands by chemiluminescence (Amersham Biosciences). For immunoprecipitation, we added antibodies against β-catenin or βPix to cytoplasmic or nuclear lysates for 2 h, followed by protein A- or protein G-agarose beads for 1 h. We washed beads thrice in PBS, released immunoprecipitated proteins from beads by boiling in 1 × sample buffer (5 min), and immunoblotted. We employed β-actin and H2A loading controls to ascertain that equal amounts of cytoplasmic and nuclear proteins, respectively, were included in the extracted protein *input*. After immunoprecipitation, as a second loading control, we immunoblotted for expression of the *bait* protein (e.g., βPix following immunoprecipitation with anti-βPix antibody); this is marked by a vertical line to the left of the images obtained from the same gel.

### Nuclear and cytosolic fractionation

As described previously^38^, we washed cells twice with ice-cold PBS, harvested cells with a rubber policeman, and lysed cells in 20 mM HEPES, pH 7.0, 10 mM KCl, 2 mM MgCl2, 0.5% Nonidet P-40, 1 mM Na3VO4, 10 mM NaF, 1 mM phenylmethanesulfonyl fluoride, and 2 µg/ml aprotinin. After a 10-min incubation on ice, we homogenized cells (20 strokes in a Dounce homogenizer) and sedimented nuclear homogenates by centrifuging at 1,500 × *g* for 5 min. Supernatants were centrifuged at 16,000 × *g* for 20 min; the resulting supernatant was the non-nuclear fraction. To remove contaminating cytoplasmic membranes, we washed nuclear pellets thrice with lysis buffer. To extract nuclear proteins, we resuspended isolated nuclei in 150 mM NaCl, 1 mM EDTA, 20 mM Tris–Cl, pH 8.0, 0.5% Nonidet P-40, 1 mM Na_3_VO_4_, 10 mM NaF, 1 mM phenylmethanesulfonyl fluoride, and 2 µg/ml aprotinin, and sonicated the material for nuclear lysis. We collected nuclear lysates after centrifugation (16,000 × *g* for 20 min at 4 °C) and electrophoresed the material on 7.5% SDS–polyacrylamide gels. We transferred proteins to polyvinylidene difluoride membranes, immunoblotted with antibodies, and detected bands by electrochemiluminescence.

### Cell transfection

We performed FLAG-βPix and transient transfection of HCT116 colon cancer cells with FLAG-βPix using Trans-It transfection 2020 reagent (Mirus, Madison, WI) following manufacturer’s instructions^38^.

### RNA interference

We transfected HT-29 and SNU-C4 cells with 50 nM siRNA targeting human β-catenin^66^. We transfected HCT116 cells with siRNA targeting human βPix: siRNA #1, 5’-GAGCUCGAGAGACACAUGGTT-3’; siRNA #2, 5’-GGAUAUUAGUGUCGUGCAATT-3’ (Ambion)^38^. Silencer Negative Control siRNA #1 (Invitrogen) was used as control. For shRNA experiments using HT-29 cells, we targeted human GIPZ viral particles against the following sequence: AGGATGAAGTTCAAGAATT (Thermo Scientific)^38^. For shRNA, we used non-silencing-GIPZ lentiviral shRNAmir (Thermo Scientific) as control.

### Fluorescence cell sorting

Applying methods described previously^67^, we seeded HT-29 cells (4.5 × 10^6^ cells) in 60-mm plates for 24 h. We serum starved cells for 24 h before adding 200 µM ACh for 10 min. After trypsinization, we fixed cells with 1% PFA on ice for 15 min and filtered them through 40-µm filters. We resuspended cells in 80% ethanol, incubated them for 4 min on ice, and washed with PBS. We blocked cells in PBS with 7% donkey serum and 0.1% TX-100 for 30 min at room temperature before adding primary anti-β-Pix and anti-activated β-catenin antibodies for 1 h at 4 °C. After washing with PBS, we incubated cells with secondary antibody (Alexa488; 30 min at 4 °C). We washed cells with PBS, stained nuclei with DAPI at room temperature (15 min) and washed again with Ca^2+^/Mg^2+^-free DPBS. We performed flow cytometry and measured intracellular fluorescence (ImageStreamX MKII high-speed imaging flow cytometer; Amnis). We collected bright field and fluorescent images (AF488 at 480–560 nm; DAPI at 435–505 nm, and AF555 at 560–595 nm) at a × 60 magnification. To determine nuclear localization of β-Pix (AF488 attached) and activated β-catenin (AF555 attached), we analyzed 5,000 gated cell singlets per sample and used IDEAS Analysis Software (Amnis) and the Similarity Morphology Feature.

### Colon cancer cell proliferation, migration, and invasion assays

For cell proliferation assays, we seeded HCT116 cells at 7 × 10^3^ cells per well and transfected cells with 50 nM control or βPix siRNA using TransIT-siQuest reagent according to the manufacturer (Mirus). After 24 h, we stimulated cells with 100 µM ACh for 48 h and measured cell proliferation using the CellTiter-Glo assay kit (Promega). For cell migration assays, we placed HT-29 cells in the upper chamber of uncoated inserts (BD Biosciences). Cells on the underside of inserts were fixed, stained (Hema 3; Fisher), and counted in five random high-power fields (HPF). We performed Invasion assays using Biocoat Matrigel Invasion Chambers (Corning) as described previously^6,52^.

### Induction of murine colon neoplasia

We treated 10- to 17-week-old *Arhgef7* CKO and littermate control male and female mice with once weekly intraperitoneal injection of 7.5 mg AOM/kg body weight for four weeks. Starting one week after the last AOM injection, 2.5% DSS was added to the drinking water for 5 days. In a separate experiment, after AOM/DSS treatment, we added bethanechol (400 µg/ml) to the drinking water for 15 weeks. Twenty weeks after the first AOM injection, investigators masked to genotype euthanized mice, harvested colons, and measured tumor number and size.

### Online cancer databases

We used *Oncomine™* (www.oncomine.org)^44^ to compare gene mRNA levels in colon cancer and normal adjacent tissue. GEPIA (gepia.cancer-pku.cn) analyzes RNA expression in tumor and normal samples from the National Cancer Institute Cancer Genome Atlas (TCGA) and Genotype-Tissue Expression (GTEx) databases^45^. *The Human Protein Atlas Program* provided information on proteins in cells, tissues, and organs^46^ and used UALCAN (ualcan.path.uab.edu) to analyze OMICS data^47^. We compared *ARHGEF7*, *CHRM3*, and *PTSG2* mRNA expression levels as described^57^.

### Statistical analysis

Unless indicated otherwise, we expressed data as mean ± SE from at least three experiments and analyzed results using Student’s two-tailed *t*-test, Mann–Whitney *U* test, one-way ANOVA, with either Tukey’s HSD post hoc or Dunn’s tests using SigmaPlot 13.0 (Systat Software, Inc., San Jose, CA) and two-tailed Fisher’s exact test using GraphPad Prism (San Diego, CA). We considered a *p*-value < 0.05 statistically significant.

### Supplementary Information

Supplementary Information.

## Data Availability

The datasets used and/or analyzed during the current study are available from the corresponding author on reasonable request.

## Abbreviations

AOM: Azoxymethane
DSS: Dextran sodium sulfate
H&E: Hematoxylin and eosin
CKO: Conditional knockout
MR: Muscarinic receptor
M_1_R: M_1_ subtype muscarinic receptors
M_3_R: M_3_ subtype muscarinic receptors
qPCR: Quantitative real-time polymerase chain reaction
RNAi: RNA interference
SD: Standard deviation
SE: Standard error
WT: Wild-type
