# Supplementary material for: Muscarinic receptor agonist-induced βPix binding to β-catenin promotes colon neoplasia
Source: Sci Rep. 2023 Oct 7;13:16920. doi: 10.1038/s41598-023-44158-8 (PMC10560271; doi:10.1038/s41598-023-44158-8)

Supplemental Materials

Muscarinic receptor agonist-induced βPix binding to β-catenin promotes colon neoplasia

Kunrong Cheng, Ahmed Chahdi, Shannon M. Larabee, Mazen Tolaymat, Margaret H. Sundel, Cinthia B. Drachenberg, Min Zhan, Shien Hu, Anan H. Said, Aaron C. Shang, Guofeng Xie, Madeline Alizadeh, Natalia Sampaio Moura, Andrea C. Bafford, Richelle T. Williams, Nader N. Hanna, and Jean-Pierre Raufman

Supplemental Table 1. PCR primers used in this work.

**Genes**	**Forward primer sequence 5'-3'**	**Reverse primer sequence 5'-3'**
**Human**		
ARHGEF7	TTCATGCGCCTGGATAAATA	CCTCTTCCGGACTTCTTGAC
CHRM3	TGGTTTGATGCTCCTACCTG	AGGACAGAGGAGTGGACCAG
c-MYC	CATACATCCTGTCCGTCCAAG	GAGTTCCGTAGCTGTTCAAGT
CCND1	AAGCTGTGCATCTACACCGA	CTTGAGCTTGTTCACCAGGA
PTGS2	AGCACTTCACGCATCAGTTT	CGCAGTTTACGCTGTCTAGC
B2M	GAGGCTATCCAGCGTACTCC	ATGGATGAAACCCAGACACA
**Murine**		
Axin2	CAGAGGGACAGGAACCACTC	TGCCAGTTTCTTTGGCTCTT
c-Myc	CAGCAGCGACTCTGAAGAAG	GACTCCGACCTCTTGGCA
CcnD1	CAACGCACTTTCTTTCCAGA	GACTCCAGAAGGGCTTCAAT
Ptgs2	AGCCTTCTCCAACCTCTCCT	CAGGGATGAACTCTCTCCGT
B2m	CCAAGACCGTCTACTGGGAT	TCTTTCTGCGTGCATAAATTG

Supplemental Figure 1

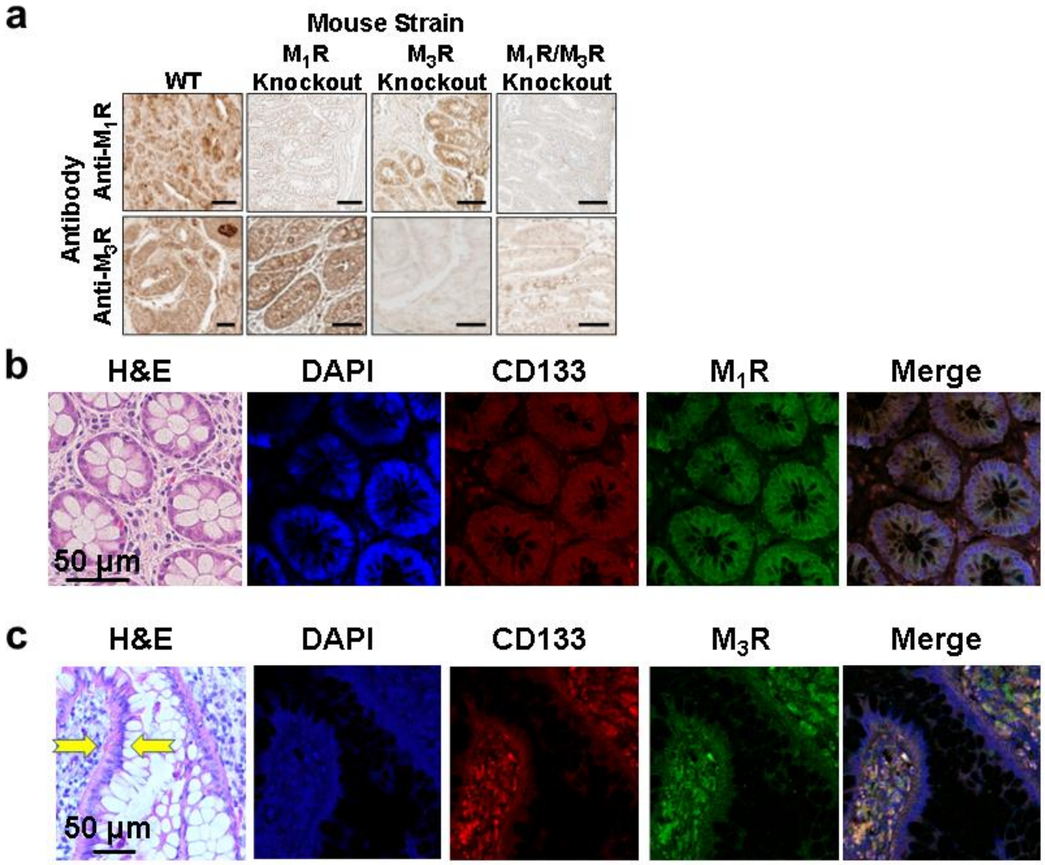

Supplemental Fig. 1. Muscarinic receptor antibody specificity. (**a**) Normal colon tissue sections obtained from WT, *Chrm1*, *Chrm3*, and dual *Chrm1*/*Chrm3* knockout mice were immunostained with anti-M_1R and anti-M_3R antibodies. Representative images confirm that M_1R and M_3R immunostaining, respectively, was absent in tissues from M_1R- and M_3R-deficient mice. Size bars, 50 µm. (**b**) Basal CD133 and M_1R staining in normal colon epithelial cells. Images show H&E (left panels) and immunofluorescent staining with DAPI (nuclear stain, blue), CD133 (red-Alexa Fluor 594), M_1R, and merged images. Size bars in H&E image, 50 µm. (**c**) Basal CD133 and M_3R staining in normal colon epithelial cells. Images show H&E (left panels) and immunofluorescent staining with DAPI (nuclear stain, blue), CD133 (red-Alexa Fluor 594), M_3R, and merged images. Arrows indicate epithelial lining. Size bars in H&E image, 50 µm.

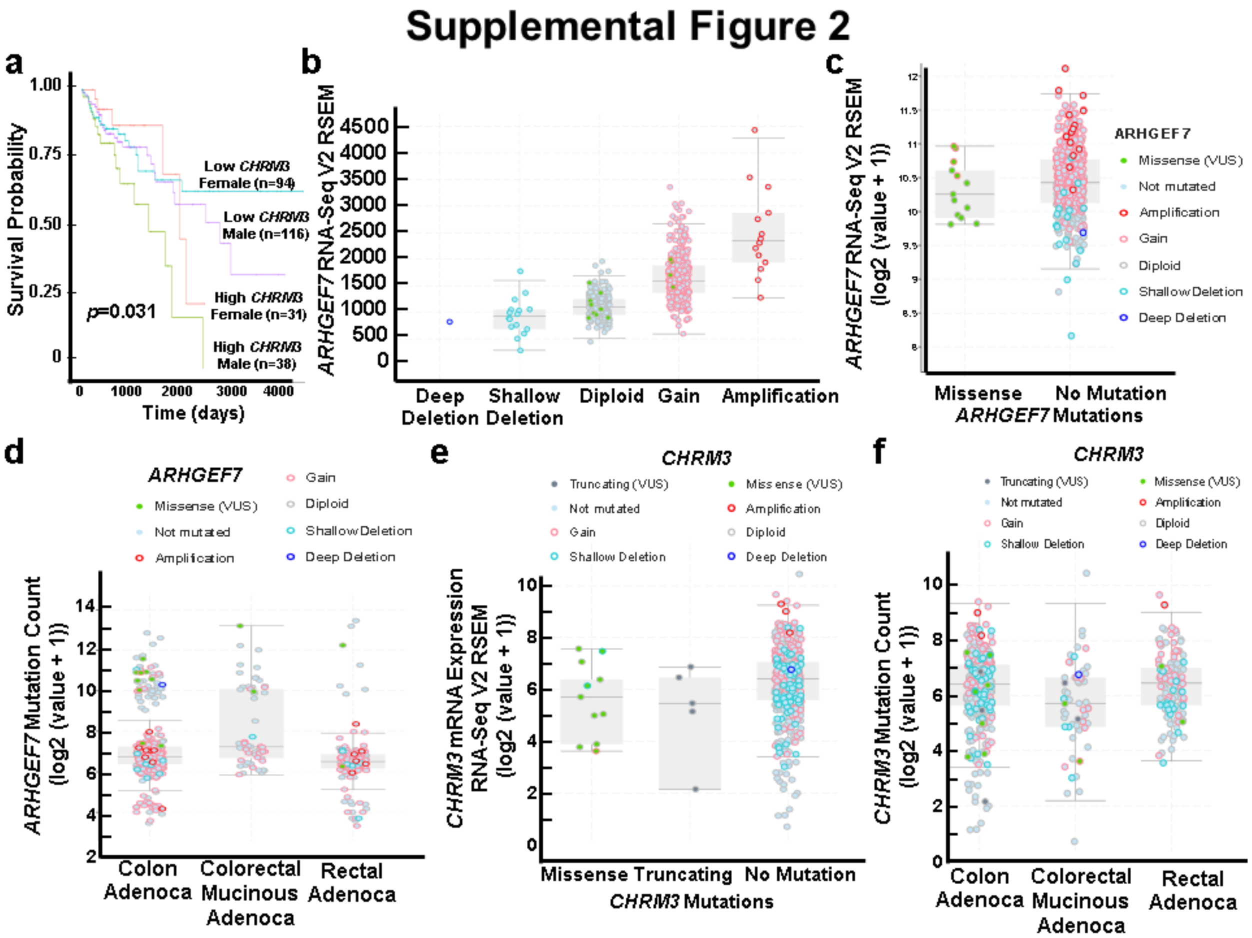

Supplemental Fig. 2. Expression levels of *CHRM3* and *ARHGEF7* in colorectal adenocarcinoma. (**a**) Effect of *CHRM3* expression levels on patient survival. Lower levels of *CHRM3* expression were associated with prolonged survival (*p*=0.031). Image downloaded from UALCAN server. Survival probability was examined by Kaplan-Meier analysis and statistical significance determined using the log rank test. (**b**) Relationship between gene amplification and transcriptional upregulation of *ARHGEF7* in CRC. (cBioPortal analysis of the TCGA PanCancer Atlas dataset, www.cBioportal.org). (**c**) *ARHGEF7* copy number alterations in colon tumor samples obtained from 526 patients (333 colon adenocarcinomas; 137 rectal adenocarcinomas; 56 mucinous colorectal adenocarcinomas) reveal 15 missense mutations in 13 patients with an overall frequency of *ARHGEF7* mutation of 2.4%. (**d**) Although there is no apparent difference in *ARHGEF7* mRNA expression between colon adenocarcinoma, colorectal mucinous adenocarcinoma, and rectal adenocarcinoma, *ARHGEF7* missense mutations were more common in colon compared to rectal adenocarcinoma. (**e**) *CHRM3* copy number

alterations in colon tumor samples obtained from 526 patients (333 colon adenocarcinomas; 137 rectal adenocarcinomas; 56 mucinous colorectal adenocarcinomas) reveal 11 missense and 5 truncating mutations in 16 patients with an overall frequency of *CHRM3* mutation of 3.0%. (**f**) There is no apparent difference in *CHRM3* mRNA expression and mutations between colon, colorectal mucinous, and rectal adenocarcinomas.

Supplemental Figure 3

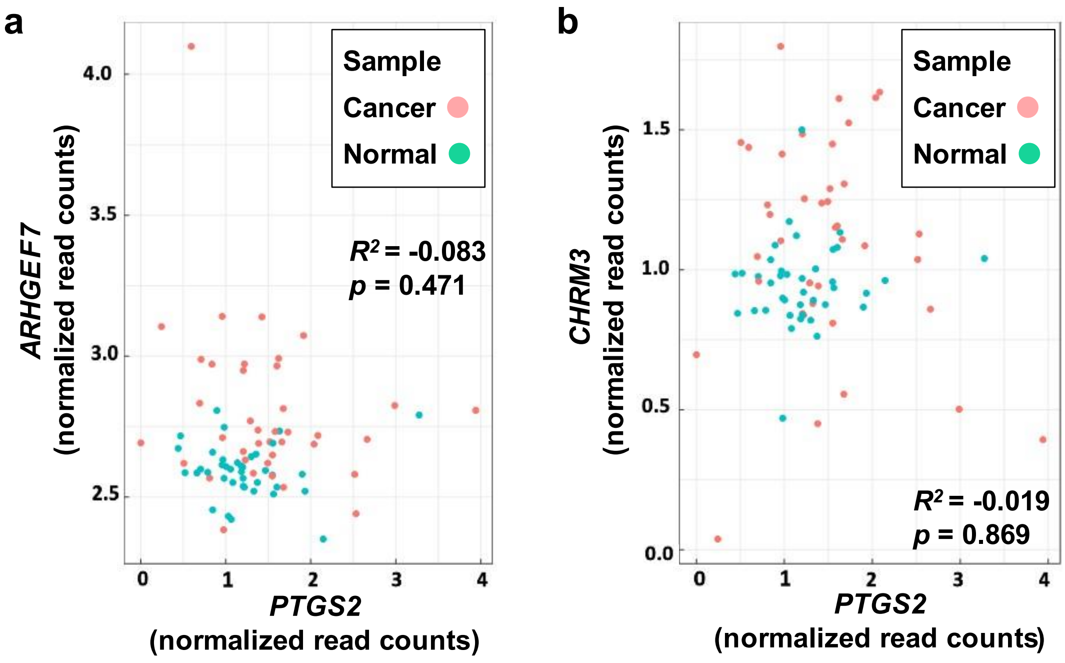

Supplemental Fig. 3. Lack of correlation between *ARHGEF7* and *CHRM3* mRNA expression levels with *PTGS2* mRNA expression levels in human colon adenocarcinomas. Univariate mixed models incorporating participant ID as a random effect were employed to compare (**a**) *ARHGEF7* and (**b**) *CHRM3* expression levels as a function of *PTGS2* levels in colon adenocarcinomas and paired adjacent normal tissue samples (n=78 for each). Values are expressed as 'normalized read counts' as described in Methods. No correlation was found between *PTGS2* and *ARHGEF7* (p=0.471) or *CHRM3* (p=0.869) mRNA expression levels.

Original uncut gels for immunoblots (the following images show the respective regions of the original blots, labelled using red boxes delineating the cropped versions shown in the manuscript figures). In red, we also indicated the proteins that was targeted for analysis. To ascertain that equal amounts of cytoplasmic and nuclear proteins were included in the extracted protein *input* for immunoprecipitation, in preliminary gels we used β-actin and H2A loading controls, respectively. Then, we immunoblotted immunoprecipitates for expression of the *bait* protein as a second loading control (e.g., immunoblotting for βPix following immunoprecipitation with anti-βPix antibody).

Fig. 2d

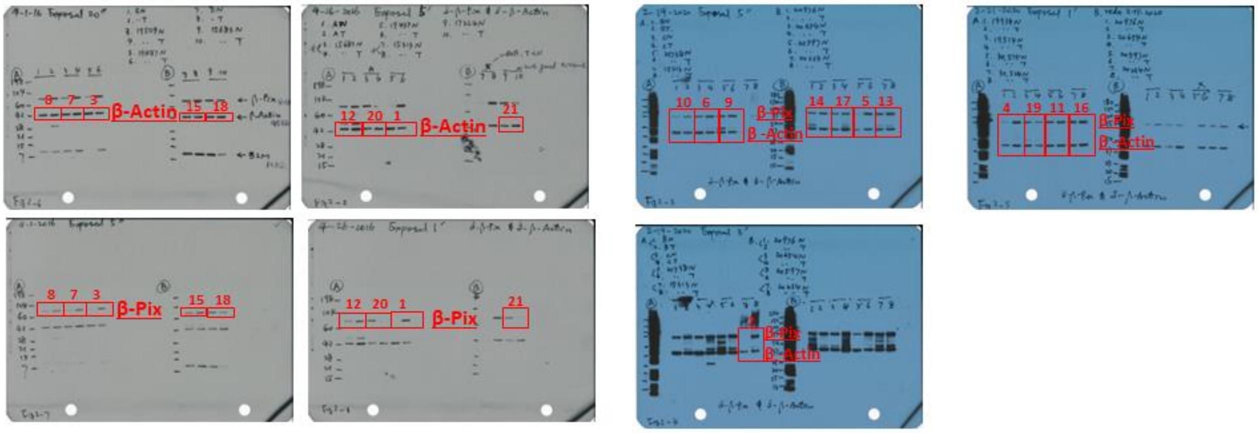

Fig. 3a

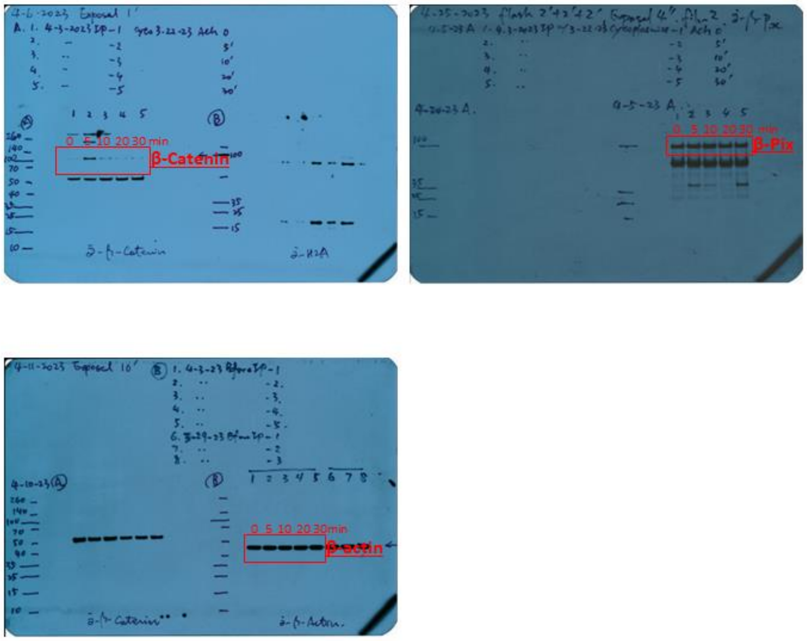

Fig. 3b

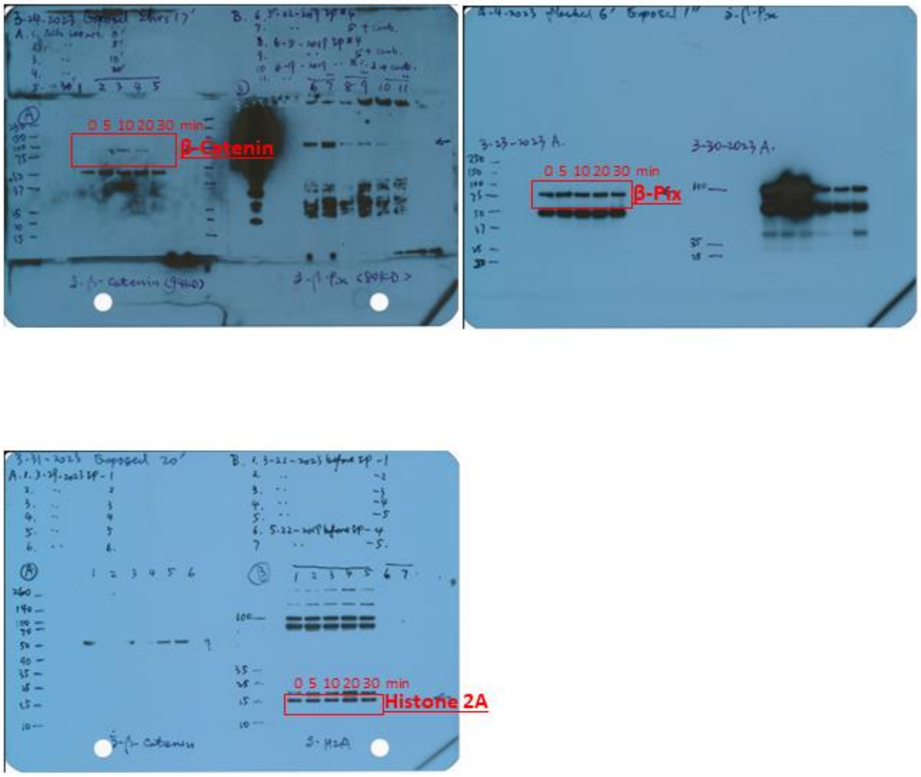
0 5 10 20 30 min
β-Catenin
0 5 10 20 30 min
β-Pix
0 5 10 20 30 min
Histone 2A

Fig. 3c

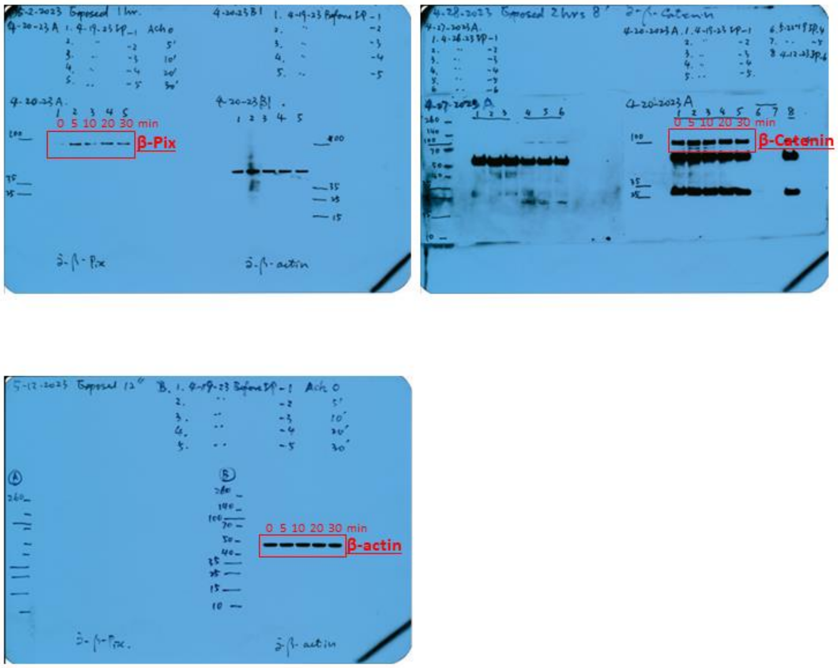
0 5 10 20 30 min
β-Pix
0 5 10 20 30 min
β-Catenin
0 5 10 20 30 min
β-actin

Fig. 3d

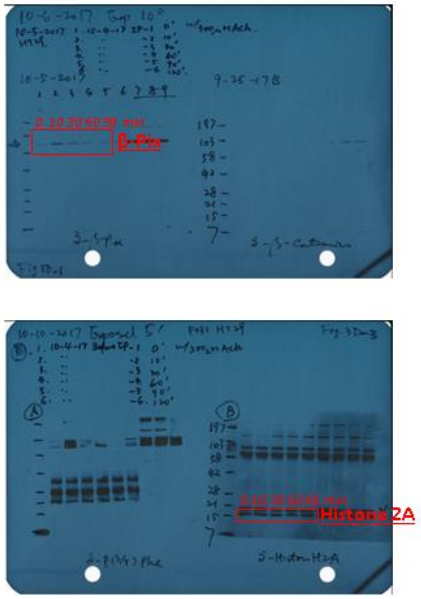
0 10 30 60 90 min
β-Pix
β-Catenin
Histone 2A

Fig. 3e

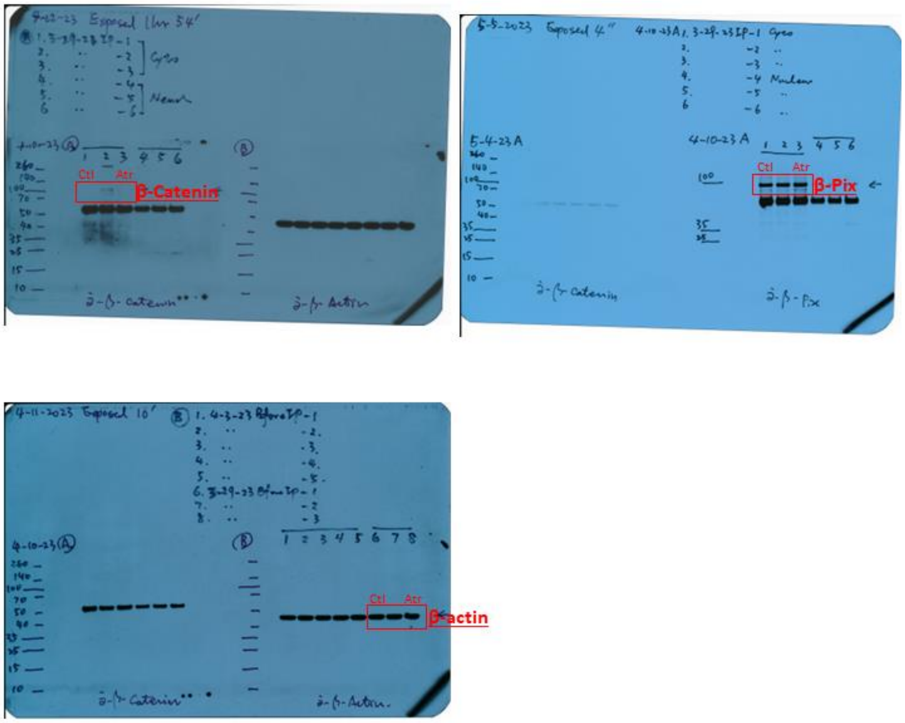
Ctl
Atr
β-Catenin
β-Pix
β-actin

Fig. 3f

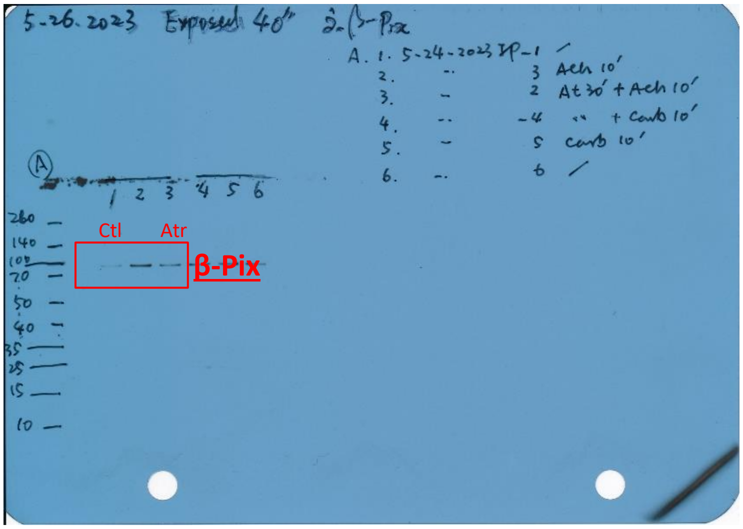

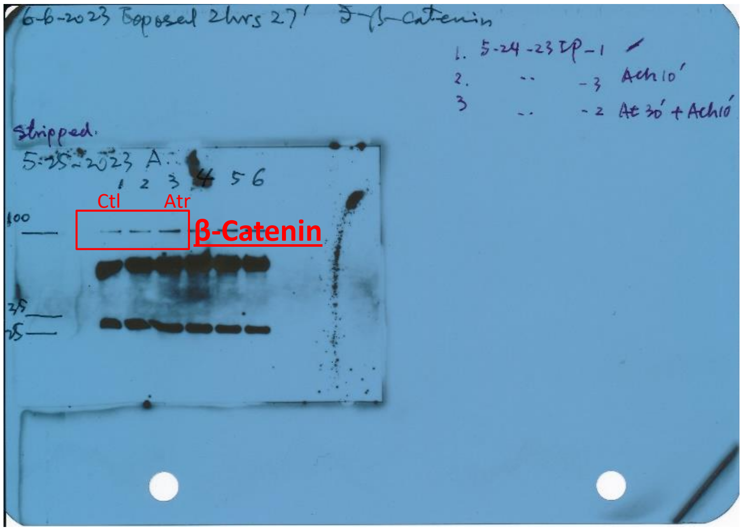

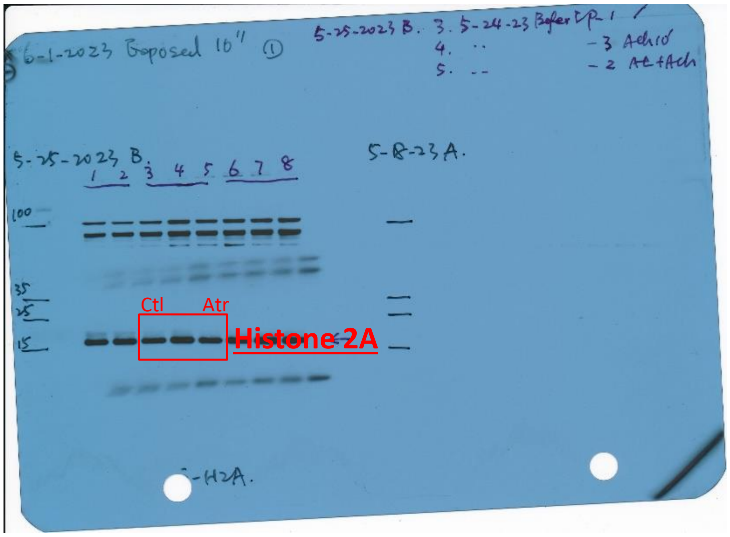

Fig. 3g

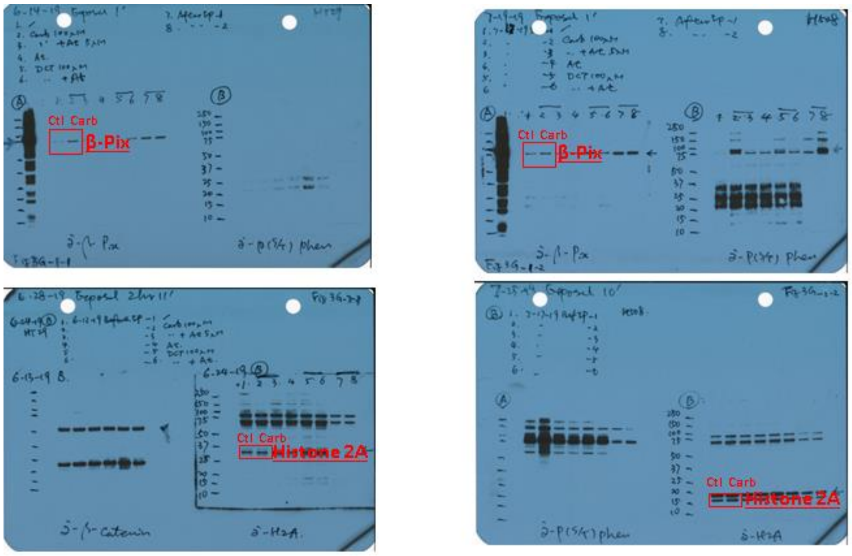
Ctl Carb
β-Pix
Ctl Carb
β-Pix
Ctl Carb
Histone 2A
Ctl Carb
Histone 2A

Fig. 3i

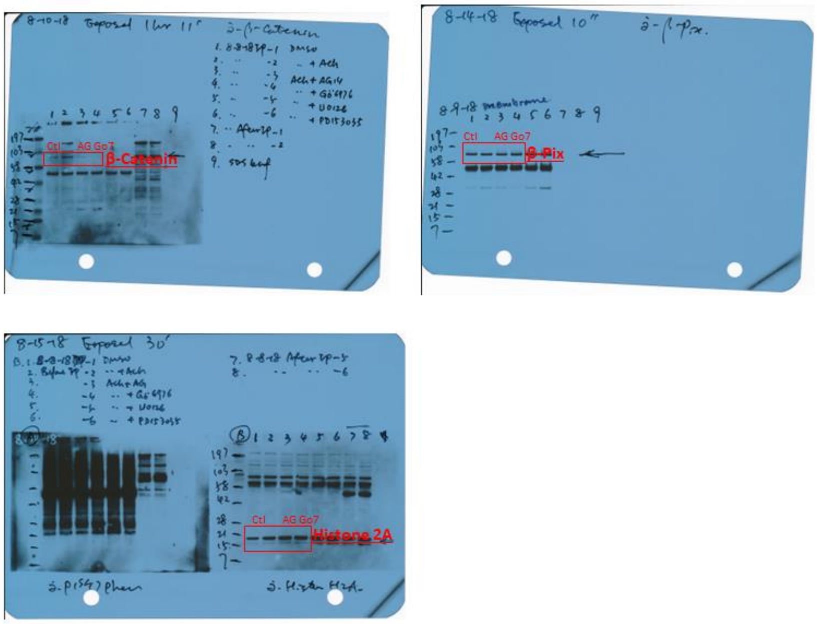
Ctl AG Go7
β-Catenin
Ctl AG Go7
β-Pix
Ctl AG Go7
Histone 2A

Fig. 3j

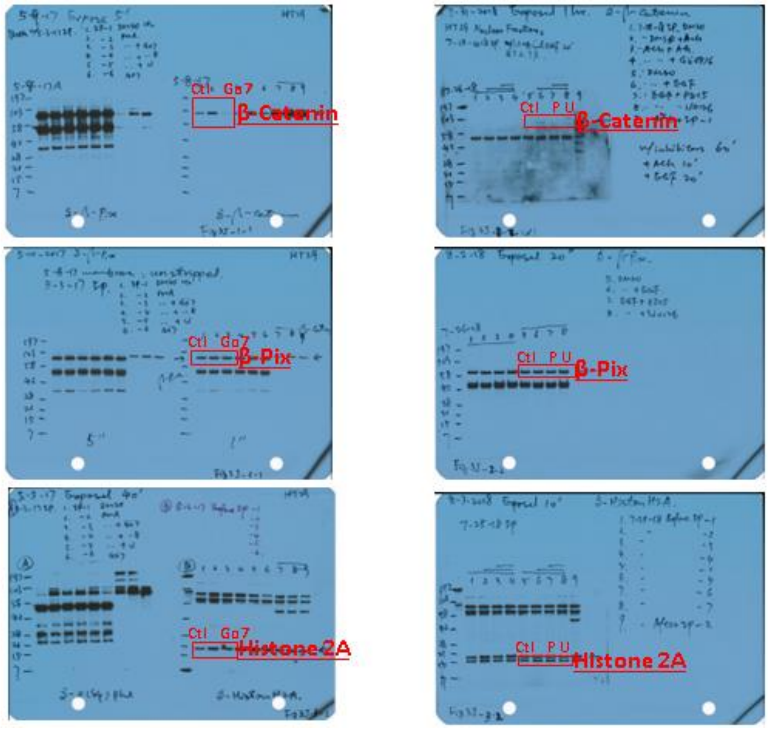
Ctl Gα7
β-Catenin
Ctl P U
β-Catenin
Ctl Gα7
β-Pix
Ctl P U
β-Pix
Ctl Gα7
Histone 2A
Ctl P U
Histone 2A

Fig. 4a

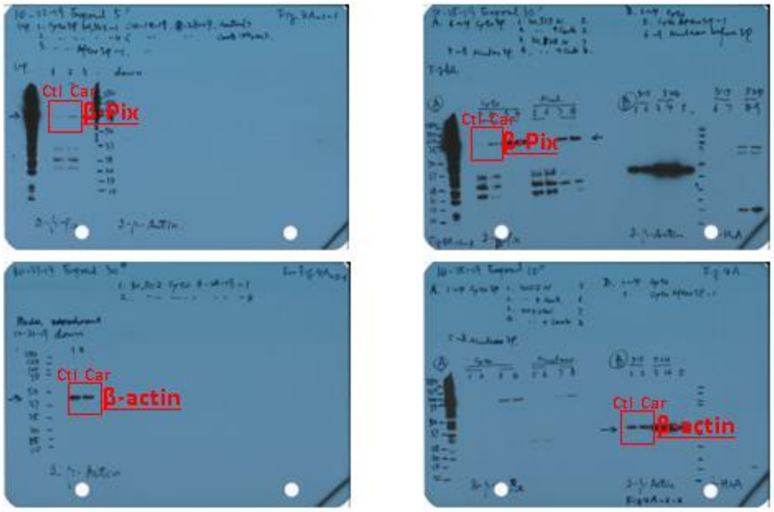
Ctl Car
β-Pix
Ctl Car
β-Pix
Ctl Car
β-actin
Ctl Car
β-actin

Fig. 4g

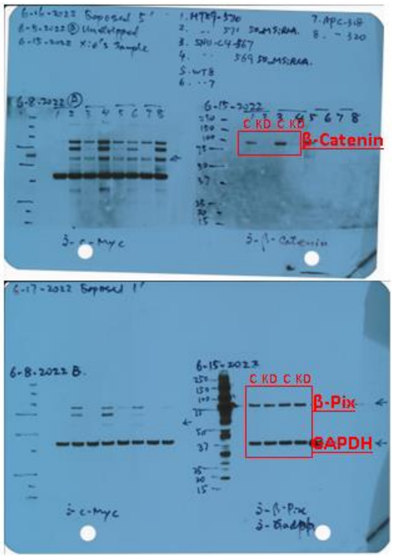
C KD C KD
β-Catenin
C KD C KD
β-Pix
GAPDH

Fig. 4h

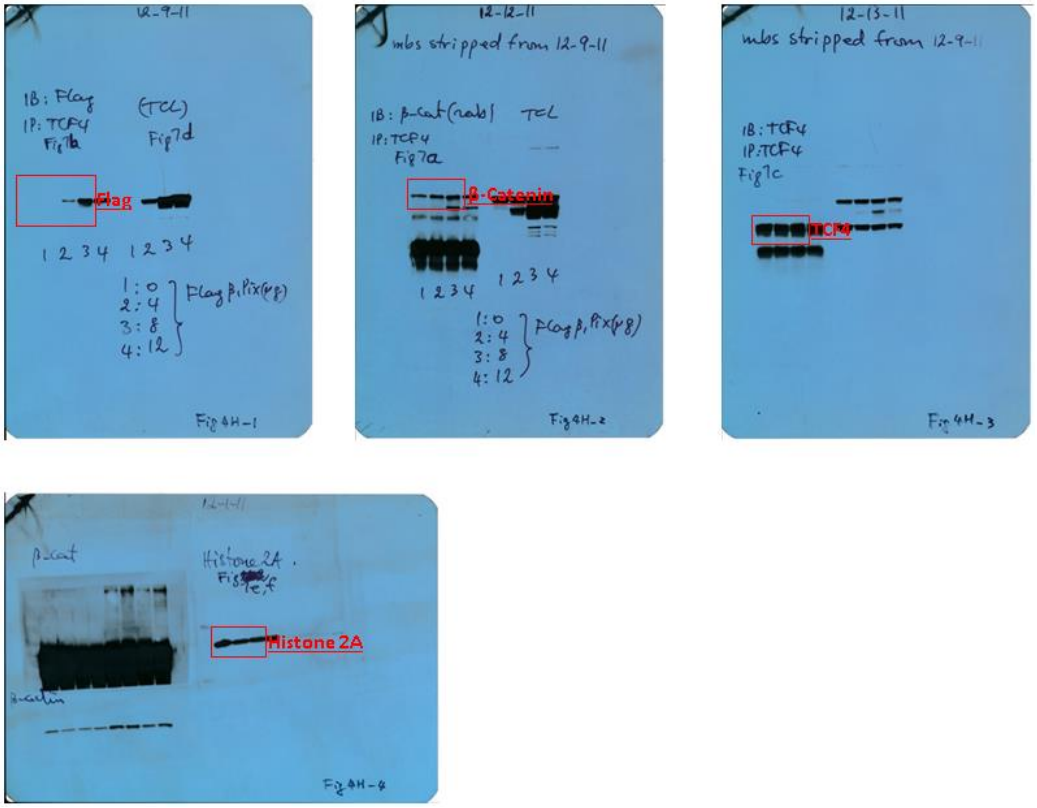
Flag
β-Catenin
TCF4
Histone 2A

Fig. 5a

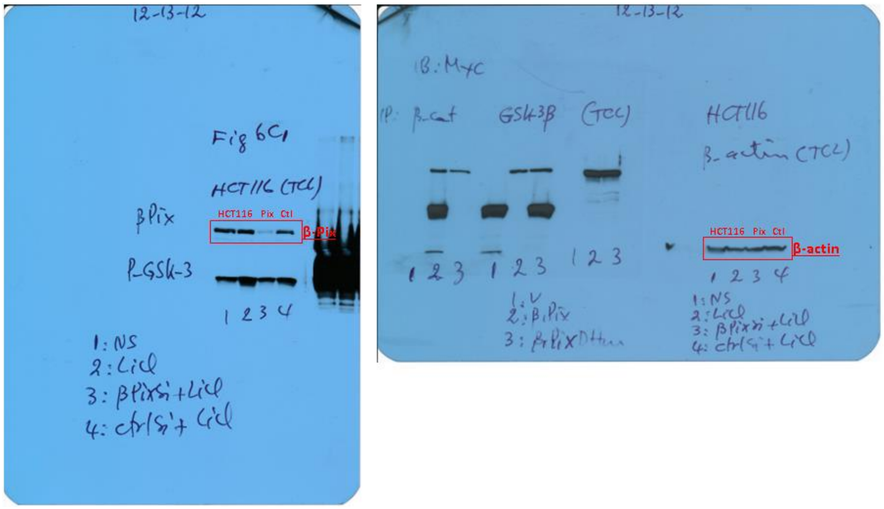

Fig. 5c

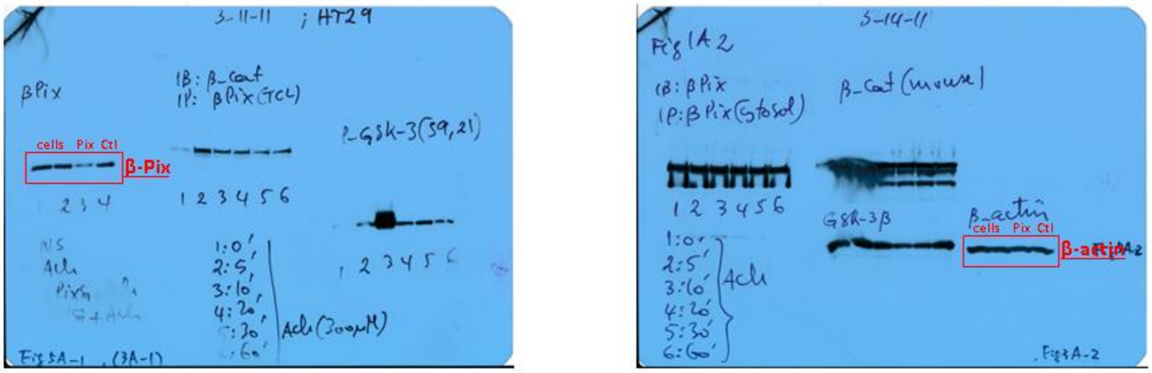

Fig. 5e

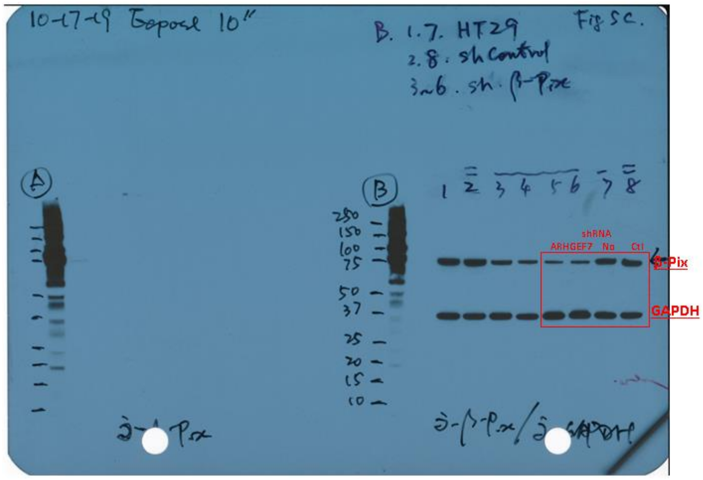

Fig. 6g

Fig. 6g_Ctl & CKO1,2

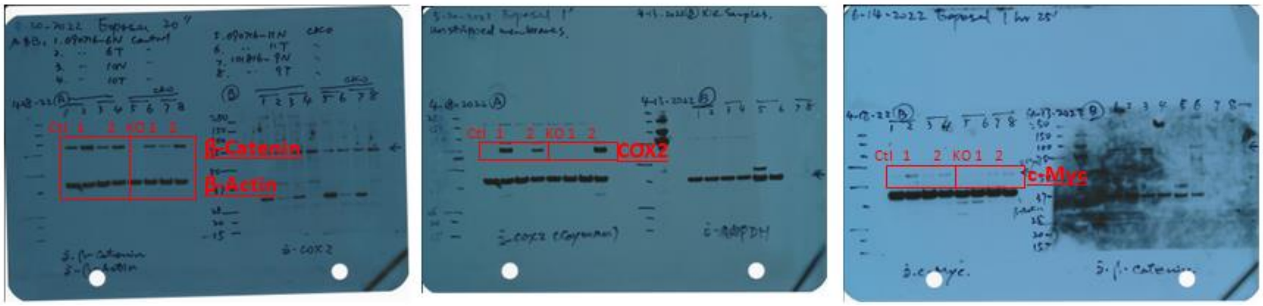

Fig. 6g_Ctl & CKO 3,4,5,6

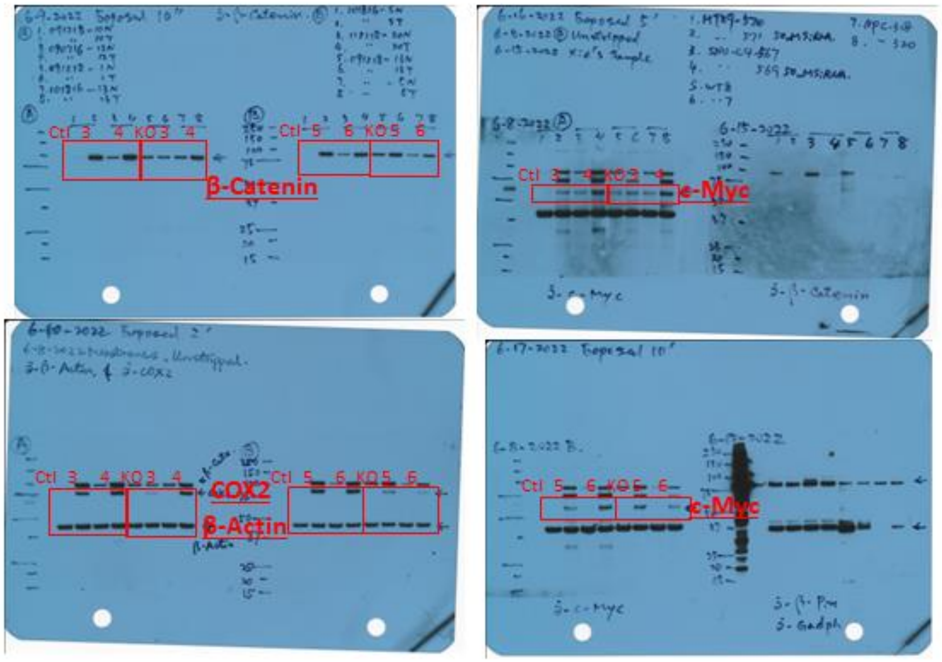

Supplement: Supplementary file 1 — Supplementary Information. [file 41598_2023_44158_MOESM1_ESM.pdf]
